# 3D chromatin remodelling in the germ line modulates genome evolutionary plasticity

**DOI:** 10.1038/s41467-022-30296-6

**Published:** 2022-05-11

**Authors:** Lucía Álvarez-González, Frances Burden, Dadakhalandar Doddamani, Roberto Malinverni, Emma Leach, Cristina Marín-García, Laia Marín-Gual, Albert Gubern, Covadonga Vara, Andreu Paytuví-Gallart, Marcus Buschbeck, Peter J. I. Ellis, Marta Farré, Aurora Ruiz-Herrera

**Affiliations:** 1 Departament de Biologia Cel·lular, Fisiologia i Immunologia, Universitat Autònoma de Barcelona, Cerdanyola del Vallès, Spain; 2 Genome Integrity and Instability Group, Institut de Biotecnologia i Biomedicina, Universitat Autònoma de Barcelona, Cerdanyola del Vallès, Spain; 3 School of Bioscience, University of Kent, Canterbury, UK; 4 Cancer and Leukemia Epigenetics and Biology Program, Josep Carreras Leukaemia Research Institute (IJC), Campus ICO-GTP-UAB, Badalona, Spain; 5 Sequentia Biotech, Barcelona, Spain; 6 Program for Predictive and Personalized Medicine of Cancer, Germans Trias i Pujol Research Institute (PMPPC-IGTP), Badalona, Spain

**Keywords:** Meiosis, Evolutionary genetics, Spermatogenesis, Genome assembly algorithms

## Abstract

Chromosome folding has profound impacts on gene regulation, whose evolutionary consequences are far from being understood. Here we explore the relationship between 3D chromatin remodelling in mouse germ cells and evolutionary changes in genome structure. Using a comprehensive integrative computational analysis, we (i) reconstruct seven ancestral rodent genomes analysing whole-genome sequences of 14 species representatives of the major phylogroups, (ii) detect lineage-specific chromosome rearrangements and (iii) identify the dynamics of the structural and epigenetic properties of evolutionary breakpoint regions (EBRs) throughout mouse spermatogenesis. Our results show that EBRs are devoid of programmed meiotic DNA double-strand breaks (DSBs) and meiotic cohesins in primary spermatocytes, but are associated in post-meiotic cells with sites of DNA damage and functional long-range interaction regions that recapitulate ancestral chromosomal configurations. Overall, we propose a model that integrates evolutionary genome reshuffling with DNA damage response mechanisms and the dynamic spatial genome organisation of germ cells.

## Abstract (web summary)

The role of genome folding in the heritability and evolvability of structural variations is not well understood. Here the authors investigate the impact of the three-dimensional genome topology of germ cells in the formation and transmission of gross structural genomic changes detected from comparing whole-genome sequences of 14 rodent species.

## Introduction

Unveiling the genomic basis of speciation is a main research priority in biology fuelled by the availability of an unprecedented large number of genomic resources. Comparative genomics of both closely and distantly related mammalian species have revealed that genomic regions implicated in structural evolutionary changes are clustered in regions more prone to break and reorganise^1–4^. In this context, evolutionary breakpoint regions (EBRs) are considered genomic regions implicated in structural evolutionary changes that disrupt genomic syntenic regions^1–4^. In searching for the origin and functional implications of this evolutionary rearrangements, research has pointed to repetitive elements as possible drivers^1,5,6^, while changes in gene expression caused by genome reshuffling may provide a selective advantage through the development of new adaptive characters specific to mammalian lineages^3,4,7–9^. Given the diversity of factors associated with EBRs it is most unlikely that sequence composition of genomes is solely responsible for genomic instability during evolution, and that the regulation of 3D genome folding is also a critical factor^8,10–12^.

Mammalian genomes are packaged into a chromatin structure, the regulation of which depends on several superimposed layers of organisation, including chromosome territories within which chromatin is organised into compartments (open/closed), which in turn consist of topologically associated domains (TADs) and DNA loops^10,13,14^. The characterisation of how chromatin conformation and DNA-protein interactions have evolved during mammalian diversification is providing a new interpretive hypothesis on the mechanism(s) responsible for the origin of genome architecture and plasticity^10,15,16^. Distant loci within the genome interact in a regulatory manner during the cell cycle^12,14,15^, affecting their ultimate function thus providing grounds for exploring the dynamics of genome composition, the evolutionary relationships between species and, in the long run, speciation. This view has been unified by the ‘Integrative Breakage Model’, an interpretative evolutionary hypothesis which posits that the permissiveness of genomic regions to reorganise can be determined by the higher-order chromatin structure^10,11^.

As with any evolutionary change of state, chromosomal reorganisations that originate in the germ line before meiosis (proliferating primordial germ cells, spermatogonia and oogonia), during meiotic division (spermatocytes and oocytes) or in post-meiotic stages (i.e., round spermatids) can be transmitted to subsequent generations. In such cases, chromosomal reorganisations can reduce gene flow and potentially contribute to speciation by the suppression of recombination in the reorganised regions between chromosomally different, but contiguous populations^16–19^. Low levels of recombination could lead to a high divergence and fixation of new mutations in these regions^20^ which, combined with the presence of genes related to species-specific evolutionary pressures may reinforce the adaptive value of EBRs in the germ line^8^. Theoretical work has suggested that heritable rearrangements would occur in genomic regions that are accessible in germ cells and/or early totipotent developmental stages^10^, highlighting the existence of a constraining role of EBRs in the germ line that needs further investigation.

Different sources of potential genomic structural alterations can arise during spermatogenesis: (i) formation and repair through homologous recombination (HR) and non-allelic homologous recombination (NAHR) of meiotically programmed double strand breaks (DSBs) catalysed by SPO11 in early stages of prophase I (i.e., primary spermatocytes in leptotene and pachytene stages)^21^, (ii) non-disjunction and meiotic drive in meiosis I and II^22^, (iii) formation and repair through non-homologous DNA end joining (NHEJ) or microhomology-mediated end joining (MMEJ) of DSBs generated in later stages of spermatogenesis (i.e., round spermatids)^23,24^ and (iv) zygotic repair of SSBs (single strand breaks) and DSBs generated by oxidative damage in mature sperm^25^. Moreover, there is fine-tuning between chromatin remodelling, architectural proteins and cell-specific gene expression during spermatogenesis^12,16,26,27^ that can affect the potential outcomes of genetic damage in the germ line. It is not known, however, which of these sources contribute most to the formation of transmissible evolutionary chromosomal reorganisations.

Here we investigate how 3D genome folding relates to the functional and the epigenetic features of evolutionary chromosomal reorganisations in the mouse germline. To do so, we (i) reconstruct ancestral rodent genomes analysing whole-genome sequences of 14 rodent species representatives of the major phylogroups, (ii) detect lineage-specific chromosome rearrangements and (iii) identify the dynamics of the structural and epigenetic properties of EBRs through mouse spermatogenesis by applying integrative computational analyses.

We find that EBRs are located in chromatin environments that become more accessible as meiosis progresses, especially in post-meiotic stages. Moreover, our results show that EBRs are devoid of programmed meiotic DSBs and meiotic cohesins in primary spermatocytes but associated with functional long-range interaction regions and sites of DNA damage in post-meiotic cells. Overall, we propose a model that integrates evolutionary genome reshuffling with DNA damage response mechanisms and the dynamic spatial genome organisation of germ cells. As such, we detect the presence of long-range interactions in spermatids recapitulating evolutionary syntenic associations present in the Muridae ancestor. Our results suggest 3D genome organisation of post-meiotic cells (i.e., spermatids) to be a major contributor to the formation of transmissible evolutionary chromosomal reorganisations.

## Results

### Patterns of chromosome rearrangements in rodents

**Fig. 1:**
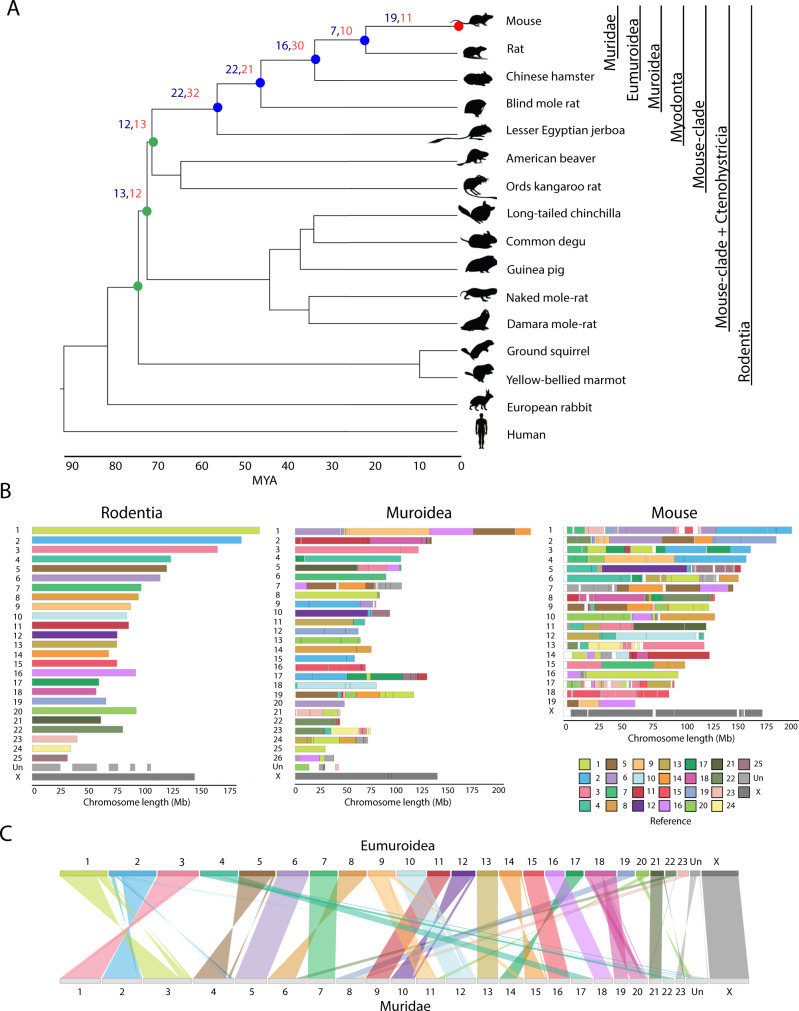
Phylogenetic tree of rodent species compared and reconstructed ancestors. **A** Phylogenetic tree constructed from the pairwise divergence times between the house mouse and each of the rodent species allows reconstructing the karyotypes for seven ancestors from Rodentia to mouse. The chromosomal rearrangements between the ancestors are shown for each node: in blue the number of inversions, in red the number of inter-chromosomal rearrangements. Green coloured dots denote ancestral lineages; blue dots represent recent ancestors (Muridae, Eumoroidea, Muroidea and Myodonta, respectively). The red depicts mouse. **B** Reconstructed ancestral karyotypes for the ancestors Rodentia, Muroidea and mouse, coloured according to Rodentia RACFs. The smaller RACFs (less than 26 Mb) are shown as an unplaced chromosome (Un). **C** Pairwise comparison between the Eumuroidea and Muridae ancestors. Each ribbon represents the syntenic fragments between the ancestors, tilted ribbons indicate inversions. Syntenic fragments are coloured according to Rodentia RACFs. Abbreviations – MYA million years ago, Un unplaced fragments.

To assess the evolutionary reshuffling of rodent genomes, we used DESCHRAMBLER^28^ with 14 Rodentia chromosome-level genome assemblies and two outgroups (human and rabbit) (Supplementary Table 1). Rodent species included in the study were representatives of the major phylogroups, with diploid numbers ranging from 22 to 72 chromosomes. We first defined the reconstructed ancestral chromosome fragments (RACFs) for seven ancestors in the rodent lineage (Muridae, Eumuroidea, Muroidea, Myodonta, Mouse Clade, Mouse Clade + Ctenohystricia, and Rodentia) (Fig. 1 and Supplementary Fig 1). After manual curation (see Methods), the final number of RACFs varied from 26 in the Eumuroidea ancestor to 35 in the Mouse clade and Myodonta ancestors (Table 1). Coverage of the mouse genome ranged from 92.92% in the Rodentia ancestor up to 97.71% in the Muridae ancestor (Table 1).

**Table 1:** Summary statistics of the reconstructed ancestral chromosome fragments (RACFs) and evolutionary breakpoint regions (EBRs) in the seven rodent ancestors and mouse.

Ancestor	No. RACFs		No. ancestral chr.	FISH^a^	Coverage of mouse genome (%)	No. of EBRs	Rate (EBR/my)
	Raw	Curated					
Mouse	–	–		–	100	54	2.58
Muridae	62	28	24	25	97.71	5	0.42
Eumuroidea	34	26	24	24	95.52	13	1.06
Muroidea	60	30	27	26	95.36	75	7.50
Myodonta	78	35	30	–	94.52	41	2.75
Mouse Clade	49	35	27	–	94.17	30	27.27
Mouse Clade + Ctenohystricia	59	33	25	–	93.66	10	5.00
Rodentia	53	31	26	25	92.92	4	0.40

^a^Reference: ^54^.

Given that our approach predicts RACFs that in some cases may not represent full ancestral chromosomes, we estimated the number of ancestral chromosomes as those RACFs ≥ 26 Mbp, the length of the smallest chromosome in the assemblies used^29^ (see Methods). Our results predicted the same number of ancestral chromosomes in Eumuroidea (haploid complement, *n* = 24), one more in the Rodentia (*n* = 26) and Muroidea (*n* = 27) ancestors, and one less in Muridae (*n* = 24) than previously reported using cytogenetics comparisons (Table 1). Despite these differences, our results recover the large majority of the syntenic associations in the Muridae ancestor previously reported using the mouse genome (*Mus musculus domesticus*, MMU) as a reference. Particularly, we were able to detect seven syntenic associations (MMU7/19, MMU5/11, MMU16/11, MMU13/15, MMU2/13, MMU1/17, and MMU17/10), with the exception of MMU12/17. The high resolution provided by our genomic comparative approach, which permitted the identification of small rearrangements allowed us to detect three syntenic associations in the Muridae ancestor (MMU5/6, MMU11/17 and MMU5/12) not described previously using cytogenetics (Supplementary Fig 1). For Eumuroidea and Muroidea ancestors, our data recovers 7/8 syntenic associations, indicating a high degree of agreement between methodologies (Supplementary Fig 1).

Using the mouse genome as a reference, our genomic approach allowed us to identify a total of 232 EBRs considering all lineages (Table 1). EBR sizes varied between 2 bp to 3.7 Mbp with a median size of 21.5 Kbp (Supplementary Fig 2). These EBRs are located in gene-dense regions (permutation test based on 10,000 permutations, z-score 7.46, *p* value 0.001) and co-localised with transposable elements, particularly ERVs (permutation test based on 10,000 permutations, z-score 4.57, *p* value 0.001, Supplementary Table 2). A total of 2524 genes were located within EBRs, and enriched in Gene Ontology (GO) terms related to immune response [134 genes, false discovery rate (FDR) 7.18E−36] and chromatin silencing (11 genes, FDR 0.00925) (Supplementary Fig 3).

Subsequently, we phylogenetically classified EBRs based on the ancestral lineage in which they occurred, ranging from 4 to 75 EBRs in the Rodentia and Muroidea ancestor, respectively (Table 1). Using the identified EBRs, we then calculated rates of rearrangement as the number of EBRs per branch length between ancestors in million years (My), ranging from 0.40 to 27.27 EBRs/My in the ancestor of all rodents and the mouse clade ancestor, respectively (Table 1 and Fig. 1). The mouse clade ancestor showed a higher chromosomal rearrangement rate than the expected average rate of chromosomal rearrangements (3.18 EBRs/My, χ^2^ test, *p* value <0.001). EBRs were further classified based on whether they demarcate inversion (*N* = 128) or non-inversion (*N* = 104) EBRs, if they are associated with a fusion or fission, and finally grouped into ancestral (*N* = 44), recent (*N* = 134) or mouse-specific EBRs (*N* = 54), whether they occurred before or after the split of Myodonta, or are uniquely present in the mouse genome (Table 1).

We then determined the number and type of chromosomal rearrangements present at each ancestor leading from the Rodentia ancestor to mouse. A total of 240 chromosomal rearrangements comprised of inter-chromosomal (fissions and fusions) (*N* = 129) and inversions (*N* = 111) were identified across seven different ancestors (Fig. 1 and Table 2). Both inter and intra-chromosomal rearrangements occurred in all ancestral lineages. Seven inversions and 10 fusion/fissions occurred between Eumuroidea and Muridae ancestor; while 22 inversions and 32 fusion/fissions characterised the evolutionary reshuffling between the Mouse clade ancestor and Myodonta (Table 2), consistent with the highest rearrangement rate in the clade (Supplementary Fig 4).

**Table 2:** Number and type of chromosome rearrangements between reconstructed rodent ancestors.

Ancestor	Inversions	Fission/Fusion	Conserved chr.	Time (my)
Muridae → Mouse	19	11	13	20.9
Eumuroidea → Muridae	7	10	14	11.8
Muroidea → Eumuroidea	16	30	6	12.3
Myodonta → Muroidea	22	21	13	10.0
Mouse clade → Myodonta	22	32	9	14.9
Mouse + Ctenohystricia → Mouse clade	12	13	14	1.1
Rodentia → Mouse + Ctenohystricia	13	12	15	2.0

Our ancestral reconstructions allowed us to characterise the genomic regions that were maintained syntenic for 73 My (million years) of rodent evolution (multispecies Homologous Syntenic Blocks, msHSBs). We identified a total of 26 msHSBs covering 11.1% of the mouse genome, with a size ranging from 513 Kbp to 45.68 Mbp with an average length of 11.61 Mbp. At least one msHSB was present in each mouse chromosome, except in MMU4, MMU5, MMU7, MMU9, and MMU14, indicating that these chromosomes are highly rearranged. A total of 5282 genes were located within msHSBs, enriched in GO terms related to mitochondrial transport (190 genes, FDR 0.006) and detection of stimulus (1372 genes, FDR 9.7e^−24^) (Supplementary Fig. 5).

### Epigenetic landscape dynamics during mouse spermatogenesis

**Fig. 2:**
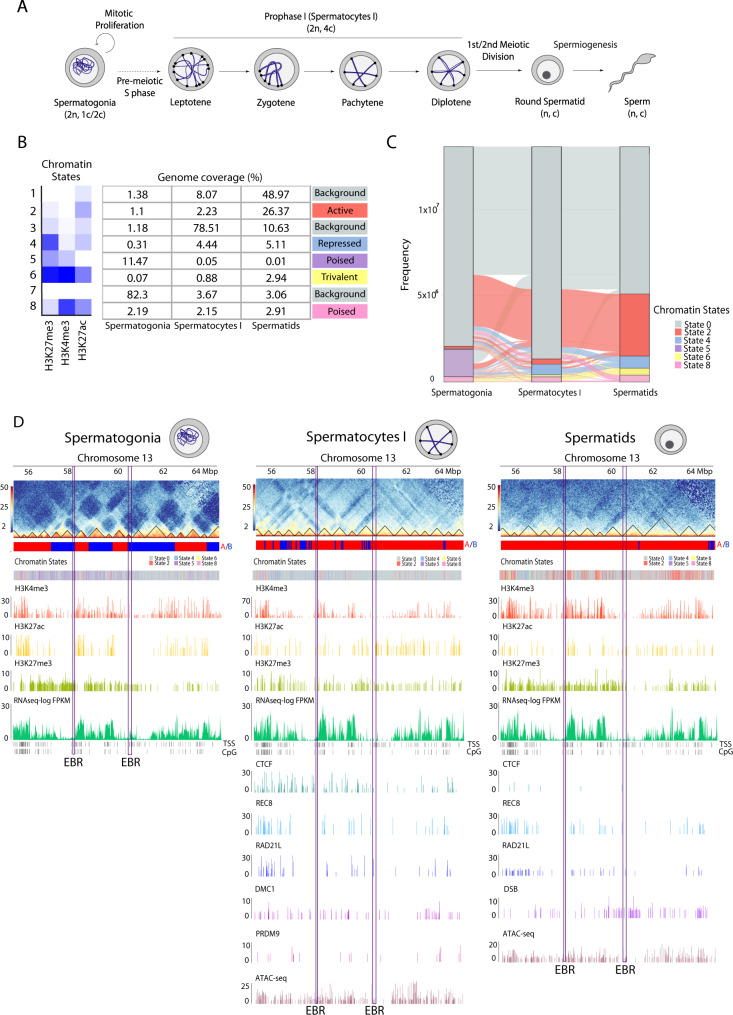
Epigenetic landscape dynamics during mouse spermatogenesis. **A** Schematic representation of mouse spermatogenesis. Adapted from^49,70^. Diploid (2n) and haploid (n) numbers are indicated for each cell type as well as the number of chromatids per chromosome (4c, 2c, or c). **B** ChromHMM chromatin states based on marks H3K27me3, H3K4me3 and H3K27ac. Numbers in the table indicate the percentage of genome coverage for chromatin states in the three cell types analysed (spermatogonia, primary spermatocytes and round spermatids). A total of 6 major chromatin states were found, including background (states 1, 3 and 7; grey), active (state 2; red), repressed (state 4; blue), poised (states 5; purple and 8; pink) and trivalent (state 6; yellow). **C** Alluvial plots representing chromatin states transitions from spermatogonia to primary spermatocytes and round spermatids. Chromatin states 1, 3 and 7 from panel (**B**) were merged into state 0 (background). **D** Chromosome 13 region-specific heatmaps at 50 kbp resolution (from 55 Mbp to 65 Mbp), for all three cell types depicting compartment signal (**A**, **B**), chromatin states, H3K4me3, H3K27ac, H3K27me3, RNA-seq (represented as log FPKM), CTCF and cohesin peaks (REC8 and RAD21L) and ATAC-seq. The genomic locations of EBRs are displayed (salmon highlight) in each cell type. Abbreviations – EBRs evolutionary breakpoint regions, FPKM fragments per kilobase of transcript per million fragments mapped.

To investigate the relationship between EBRs and chromatin structure of mouse male germ cells, we first analysed the landscape of the higher-order chromatin organisation during spermatogenesis (Fig. 2A). To that aim we re-analysed available chromatin epigenetic data^30^ for spermatogonia (pre-meiotic), primary spermatocytes (meiotic) and round spermatids (post-meiotic) using three histone marks: active associated histone modifications H3K4me3 and H3K27ac, and one repressed associated histone modification (H3K27me3) (Supplementary Table 3). In order to avoid methodological bias, we used available ChIP-seq datasets for all three cell types (spermatogonia, primary spermatocytes and round spermatids) retrieved from the same experimental study (see Methods). The percentage of the genome covered by each histone varied slightly between the cell types. H3K4me3 ranged from 1.3 to 4.6% in spermatogonia and round spermatids, H3K27me3 from 1.7% to 3.8% while H3K27ac from 1.1% to 1.3% of the mouse genome, respectively.

Using ChromHMM, we created a chromatin state model at 200 bp resolution defined by eight different chromatin states (from E1 to E8) in spermatogonia, primary spermatocytes and round spermatids (Fig. 2B and Supplementary Table 4). States E1, E3 and E7 were dominant, covering 48.9%, 78.5% and 82.3% of the mouse genome, in round spermatids, primary spermatocytes and spermatogonia, respectively. As all three chromatin states E1, E3 and E7 had low coverage of histone marks they were classified as background state (E0) (Fig. 2B). Active state E2 (enriched in H3K27ac), increased from a coverage of 1.1% of the genome in spermatogonia to 26.4% in round spermatids (Fig. 2B). State E4, however, was dominated by the repressive chromatin mark H3K27me3, spanning between 0.31% and 5.11% of the genome in spermatogonia and round spermatids, respectively. As for poised chromatin (states E5 and E8 with both H3K27me3 and H3K4me3 marks) it covered from 13.7% in spermatogonia to 2.92% in round spermatids; while state E6 (labelled as trivalent chromatin showing all three histone marks) covered 0.07% to 2.94% in spermatogonia and spermatids (Fig. 2B).

To assess the dynamics of chromatin state transitions throughout spermatogenesis we compared the transition of chromatin states from spermatogonia to spermatocytes and then to round spermatids for a given genomic region (Fig. 2C). This allowed us to investigate which regions of the mouse genome get activated or repressed during spermatogenesis. To do so, genomic regions at 200 bp resolution defined by six different chromatin states (E0, E2, E4, E5, E6 and E8) were compared between the three cell types (spermatogonia, primary spermatocytes and round spermatids). This gave us a total of 192 combinations of cell type and chromatin state, with 34 combinations covering overall >98% of the genome with at least 0.1% each. The trivalent state with all three histone marks (E6-E6-E6) was also included, representing a coverage of 0.029% of the mouse genome and making a total of 35 combinations studied (Fig. 2C, Supplementary Fig 6 and Supplementary Table 5).

When comparing the percentage of each mouse chromosome coverage for each of the 34 most frequent chromatin state combinations, we detected that autosomes have different coverage of the three-cell type states than the sex chromosomes, with state E0-E0-E0 having the largest coverage ranging from 45.43% on chromosome 11 to 62.24% on chromosome 3 (Supplementary Fig 6). For the sex chromosomes, state E0-E0-E2 had the largest coverage (44.71% on chromosome X and 50.76% on chromosome Y). State E5-E0-E0 was lower on the sex chromosomes (X:1.43% and Y:0.31%) compared to an average of 6.51% on the autosomes. State E5-E0-E2 decreased on chromosome Y with 0.4% coverage compared to an average of 2.3% on the autosomes. Instead, state E0-E0-E8 increased on chromosome Y at 3.44% compared to an average of 1.4% on the autosomes.

We detected that most of the genome (54.45%) remained in the same background chromatin state (E0-E0-E0) throughout spermatogenesis (Supplementary Fig 6). This contrasted with the small proportion of the genome that is maintained active (0.084% in E2-E2-E2), poised (0.20%, E8-E8-E8 and E5-E5-E5 < 0.001%), trivalent (0.029% E6-E6-E6) or repressed (0.12%, E4-E4-E4) in all three cell types. Remarkably, 41.09% of the genome changed chromatin state during spermatogenesis, with E0-E0-E2 being the most common transition (21.9% coverage), followed by E5-E0-E0 (6% coverage). During spermatogenesis, 25.8% of the genome became active, whereas only 4.49% and 1.94% transitioned to repressed or poised states (Fig. 2C). In contrast, a total of 2.56% of the mouse genome became trivalent in spermatids. As expected, both X and Y chromosomes are enriched in ‘closed’ chromatin states (Supplementary Fig 6) as they are subjected to meiotic sex chromosome inactivation (MSCI) during prophase I and post-meiotic sex chromatin (PMSC) in round spermatids^12,31^.

### EBRs associate with open chromatin environments activated during meiosis

We next integrated the genome positions of EBRs with chromatin states and structural datasets including Hi-C data, CTCF, meiotic cohesins (REC8 and RAD21L), CpG islands, transcription start sites (TSS), ATAC-seq and RNA-seq (see Methods) (Fig. 2D). In particular, we analysed 3D genome folding dynamics (A/B compartments and TADs) using published Hi-C maps generated for spermatogonia, primary spermatocytes and round spermatids^12^ and compared with the dynamics of the epigenetic landscape dynamics (previous section). Moreover, CTCF and meiotic cohesins binding sites were included for primary spermatocytes and round spermatids^12^. Genome-wide associations of different datasets were evaluated using permutation tests (see Methods).

**Fig. 3:**
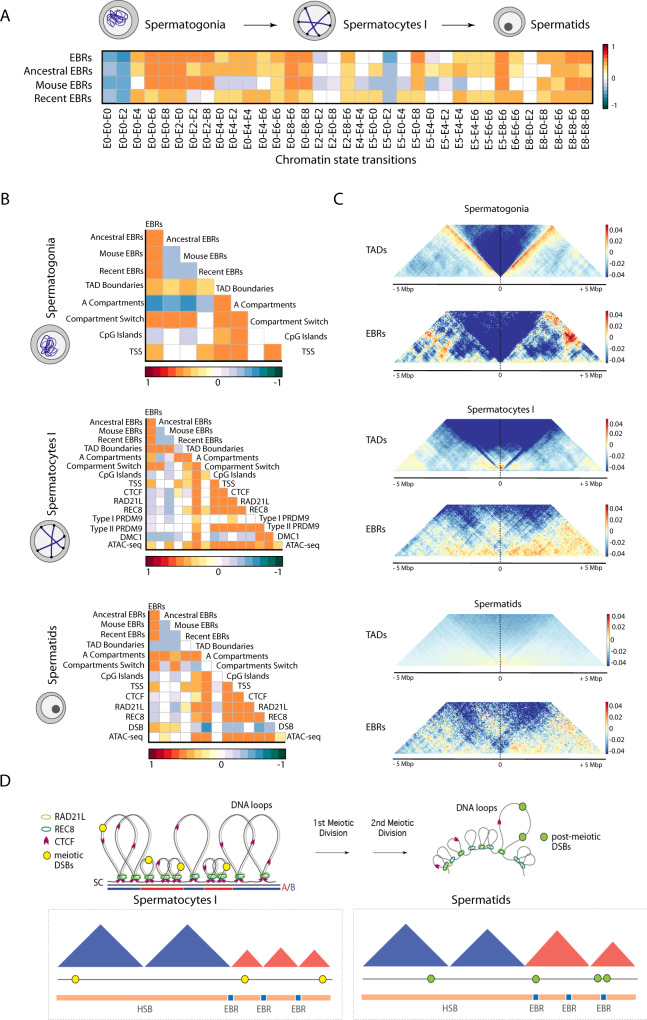
Multi-comparison analysis. **A** Heatmaps obtained by regioneR (multicomparison) displaying correlations between different EBRs (ancestral, recent and mouse specific) and chromatin state transitions between chromatin states (E) in spermatogonia, primary spermatocytes and round spermatids. **B** Heatmaps obtained by regioneR (multicomparison) displaying correlations between different EBRs (ancestral, recent and mouse specific) and, TAD boundaries, A compartments, compartment switch (from A to B and viceversa), CpG islands, transcription start sites (TSS) in the three cell types. CTCF, cohesins (RAD21L and REC8) and ATAC-seq were included for both primary spermatocytes and round spermatids. Primary spermatocytes also included PRDM9 sites (Type I and II) and DMC1 sites. Round spermatids included post-meiotic DSBs. **C** Interaction metaplots depicting insulator scores for TADs and EBRs in each cell type. **D** Working model depicting the disposition of the genome folding (DNA loops and compartments) in relation to cohesins, CTCF, meiotic DSBs and EBRs. In the case of primary spermatocytes DNA loops protrude out of the chromosomal axes with meiotic DSBs occurring inside TADs in A compartments; EBRs are associated with TAD boundaries. In the case of round spermatids, EBRs are associated with post-meiotic DSBs inside TADs in A compartments. Abbreviations – EBRs evolutionary breakpoint regions, TADs topological associated domains, DSBs double strand breaks, FPKM fragments per kilobase of transcript per million fragments mapped.

We first studied the epigenetic landscape of EBRs, by assessing the co-location of the 35 three-cell type states with the genomic positions of EBRs using a multi-association permutation test (see Methods). Remarkably, EBRs are negatively associated (permutation test based on 10,000 permutation, normalised z-score −0.05, *p* < 0.05) with the background state (E0-E0-E0), but highly associated (permutation test based on 10,000 permutation, normalised z-score > 0.01, *p* < 0.05) with active or poised chromatin (Fig. 3A and Supplementary Fig 7). This association was stronger with states that transition to E6 and E8 in spermatids (normalised z-score > 0.05, *p* < 0.05), particularly with those EBRs that occurred in the mouse lineage, suggesting that EBRs occur in chromatin environments prone to rapid change during spermatogenesis.

To further investigate labile chromatin landscapes, we analysed the gene content of those three cell type states associated with EBRs. A total of 10,925 unique protein-coding genes were present in E6 and E8 regions in spermatids. GO enrichment analysis (≥1.5-fold enrichment and FDR < 0.5) identified GO terms related to protein localisation to cell junction and protein dephosphorylation (1.79 and 1.55 fold) as well as dendrite development and regulation of organelle assembly (1.57 and 1.51 fold).

At the gross structural level, EBRs were associated with regions that altered their state during spermatogenesis (all associations based on multiple permutation test based on 10,000 permutations, normalised z-score > 0.01, *p* < 0.05) (Fig. 3A and Supplementary Fig 7). In particular, EBRs are associated with the ‘closed’ B compartment in pre-meiotic spermatogonia, but with the ‘open’ A compartment in meiotic spermatocytes and post-meiotic spermatids (Fig. 3B and Supplementary Fig 7). Consistent with this, EBRs are associated with ‘closed’ chromatin environments (E0, E4, E5) in spermatogonia and ‘open’ chromatin environments (E2, E6, E8) in both primary spermatocytes and round spermatids. At a finer structural level, too, EBRs are associated with regions that undergo structural remodelling, being associated with TAD boundaries in spermatogonia and spermatocytes, but located within TADs in round spermatids (Fig. 3B and Supplementary Fig 7). Although EBRs were associated with TSS, these regions do not present high levels of expression in spermatogonia but are expressed more highly in round spermatids (Mann–Whitney test, *p* < 2.2e−16) (Supplementary Fig 8). Overall, our results suggest that EBRs localise preferentially in genomic regions that become accessible as spermatogenesis progresses. Furthermore, evolutionary rearrangements should not disrupt TAD structures in spermatogonia or spermatocytes but may do so in round spermatids.

### Chromosome rearrangements do not disturb meiotic chromosomal architecture in prophase I

Next, we investigated whether the meiotic chromosomal architecture had an influence on the distribution of EBRs in primary spermatocytes. To do so, it is important to consider that meiotic chromosomes are organised into DNA loops anchored to chromosome axes formed by the synaptonemal complex (SC) during prophase I, a proteinaceous structure with a zipper-like morphology that mediates the synapsis of homologous chromosomes^32^. The SC establishes the context in which synapsis and recombination between homologs take place, joining sister chromatids within the lateral components of the SC axis by meiotic cohesins, such as REC8^33^ and RAD21L^34^. Importantly, while meiotic DSBs can occur at DNA loops, they are repaired in the context of the chromosomal axes. As chromosomal axis length is inversely correlated with the size of chromatin loops emerging from the SC^35^, the number and distribution of DSBs per chromosome is linked to the structural organisation of the genome during meiosis^22,36^.

In searching for the evolutionary plasticity of meiotic chromosomal architecture we conducted permutation tests (based on 10,000 permutations) to evaluate the association between EBRs and the genomic position of DMC1 and PRDM9 sites together with different structural meiotic features such as meiotic cohesins (RAD21L and REC8) in primary spermatocytes. Importantly, we detected that EBRs were negatively associated with DMC1 and PRDM9 sites (Fig. 3B). EBRs were likewise negatively associated with meiotic cohesins (RAD21L and REC8) in both primary spermatocytes and round spermatids (Fig. 3A). Even though EBRs correlated with ‘open’ chromatin environments in primary spermatocytes (i.e., E0-E6-E6 and E8-E8-E6) (Fig. 3A), these regions were not associated with cohesins (Supplementary Table 6). As cohesins are necessary structural parts of the DNA loops attached to the chromosomal axes and the formation and repair of DSBs are tightly regulated during meiosis our results suggest the presence of purifying selection for the occurrence of large scale chromosomal reorganisations in the germ line (Fig. 3C, D). These results are in line with previous observations of a reduction of recombination rates in EBRs^3,8,18^.

### EBRs associate with DNA damage in post-meiotic cells

As evolutionary rearrangements were found preferentially associated with genomic regions that become accessible as spermatogenesis progresses, we subsequently analysed the occurrence of EBRs in the context of post-meiotic cells (i.e., round spermatids). Spermatids are haploid cells with highly compacted genome (most of the histones are replaced by protamines), as such they are subjected to unique mutational pressures. These include DSBs formed (potentially as the result of topoisomerase II activity) to relieve DNA helix torsion. As round spermatids lack a template for homology directed repair, DSBs must be repaired by error-prone methods such as NHEJ or MMEJ^23,37^.

Using publicly available data (see Methods), we identified a total of 151,732 DSB hotspots in spermatids (from now on post-meiotic DSBs), covering 1.49% of the mouse genome. Regions containing post-meiotic DSBs ranged from 146 bp to 6662 bp, with a mean and a median of 267 bp and 213 bp, respectively. The location of these post-meiotic DSBs in the mouse genome correlated with chromosome size (Pearson correlation, *r*^2^ = 0.68, *p* = 0.00074), with longer chromosomes accumulating more DSBs than shorter ones, except in the chrY (3.51% coverage) (Supplementary Fig 9). Moreover, post-meiotic DSBs were also associated with repeat content in each chromosome (Pearson correlation, *r*^2^ = 0.78, *p* = 0.00003). Specifically, post-meiotic DSBs co-localised with transposable elements L1Md_T and L1Md_A (GAT analysis, 1000 permutations with 12.6- and 11.1-fold, *p* = 0.001).

At the epigenetic and structural level, we detected that post-meiotic DSBs tend to occur in genomic regions depleted of epigenetic modifications (state E0-E0-E0). That is, DSBs tend to be excluded from active or poised epigenetic landscapes in round spermatids (Supplementary Fig. 7). Also, post-meiotic DSBs were negatively associated with A-compartments (multiple permutation test based on 10,000 permutations, normalised z-score −0.06, *p* < 0.05) and TAD boundaries in round spermatids (normalised z-score −0.07, *p* < 0.05). Remarkably, post-meiotic DSB hotspots are positively correlated with EBRs (normalised z-score 0.06, *p* < 0.05, Fig. 3B and Supplementary Fig. 7), suggesting that EBRs are located in the subset of DSBs that occur in open chromatin in round spermatids. Therefore, our results indicate that transmissible genomic rearrangements preferentially occur within accessible genomic regions that suffer DNA damage in post-meiotic cells (Fig. 3D).

### Functional features of post-meiotic cell-specific long-range genomic interactions

**Fig. 4:**
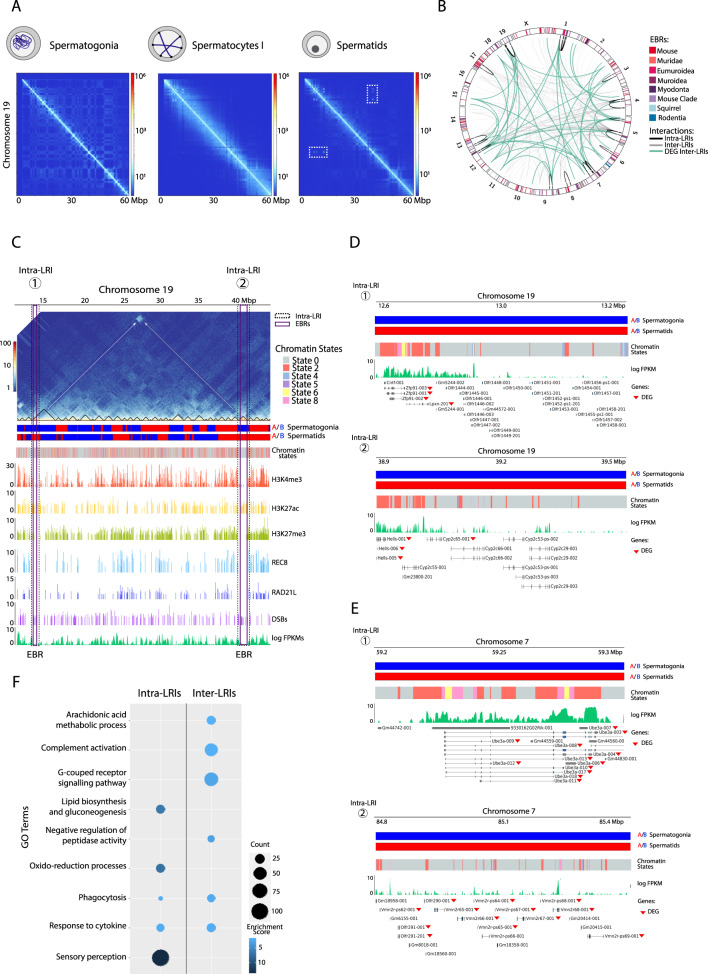
Long-range interactions in post-meiotic cells. **A** Iterative correction and eigenvector decomposition (ICE)-corrected Hi-C matrices for chromosome 19 at a 100-kbp resolution for the cell types analysed. Deep blue lines indicate non-mapped bins. Long-rage interactions are highlighted (yellow square) in round spermatids. **B** Circos plot of the mouse genome depicting the different types of EBRs identified (Mouse, Muridae, Eumuroidea, Muroidea, Myodonta, Rodentia, Mouse Clade and Mouse Clade + Ctenohystricia specific) and long-rage interactions (LRIs). LRIs containing differentially expressed genes (DEG) are shown in green. **C** Representative example of intra-chromosomal long-range interactions across a specific region of chromosome 19 (from 14 to 45 Mbp) in mouse round spermatids. Different genomic features are displayed: A/B compartments (in spermatogonia and round spermatids), chromatin states, H3K4me3 peaks, H3K27ac peaks, H3K27me3 peaks, RNA-seq (represented as log FPKM), CTCF peaks and cohesin peaks (REC8 and RAD21L). The genomic locations of EBRs (salmon highlight) and intra-chromosomal long-range interactions (square) are displayed. **D** Zoom-in of intra-chromosomal long-range interactions detected in chromosome 19, displaying A/B compartments, chromatin states, RNA-seq (represented as log FPKM), and genes from NCBI Ref Seq annotation. Differentially expressed genes (DEG) are shown (red arrow head). **E** Zoom-in of intra-chromosomal long-range interactions detected in chromosome 7, displaying A/B compartments, chromatin states, RNA-seq (represented as log FPKM), and genes from NCBI Ref Seq annotation. Differentially expressed genes (DEG) are shown (red arrow head). **F** Gene Ontology Enrichment Analysis (GOEA) of genes found in intra-chromosomal and inter-chromosomal long-range interactions (LRIs) in round spermatids. Only significant gene ontology (GO) terms with an DAVID enrichment cluster score (ES) > 1.3 are shown, ES is based on a FDR adjusted *P* value (Fisher’s exact test, two-sided). Abbreviations – EBRs evolutionary breakpoint regions, LRIs long-ranged interactions, GO gene ontology, FPKM fragments per kilobase of transcript per million fragments mapped.

A refined analysis of chromosomal-specific Hi-C maps revealed the existence of genomic regions participating in cell-specific long-range intra-chromosomal interactions in round spermatids that were absent in spermatogonia and primary spermatocytes. We dubbed these regions ‘intra-LRIs’ (intra-chromosomal long-ranged interactions) and detected the presence of 36 intra-LRIs specific to round spermatids distributed across chromosomes 1, 3, 4, 5, 7, 9, 12, 13, 17 and 19 (Fig. 4 and Supplementary Table 7) ranging in size from 0.5 Mbp to 2.05 Mbp (mean size = 0.8 Mbp). The average distance between intra-LRIs was of 24.2 Mbp, and the interactions were non-transitive, i.e. the presence of interactions A ↔ B and A ↔ C does not imply a direct interaction B ↔ C, and a single “hub” intra-LRI could interact with two or more “spoke” intra-LRIs in the same chromosome (Supplementary Table 8).

To explore the functional role of genes located within intra-LRIs in round spermatids, we analysed the GO terms contained in these regions. Out of the total 691 genes located in intra-LRIs, 41% (*n* = 283) were protein-coding genes, whereas non-coding RNAs represented 34% of the total, followed by pseudogenes (20%) and lncRNAs (1.6%). The remaining 3.4% of the genes contained in intra-LRIs were non-annotated transcripts. When analysing GO terms, we detected that intra-LRIs were enriched (*p* value < 0.05 and enrichment score (ES) > 1.3, see Methods) for genes related with sensory perception (ES = 13.6), lipid biosynthesis and gluconeogenesis (ES = 6.27), oxido-reduction processes (ES = 6.97), response to cytokines (ES = 1.94) and phagocytosis (ES = 1.74) (Supplementary Data 1). Additionally, 33% of these genes were members of relevant superfamilies, such as serpines, zinc finger proteins, olfactory receptors and vomeronasal receptors. Considering differentially expressed genes (DEG) in spermatids when compared to spermatogonia, primary spermatocytes and sperm, GO terms in intra-LRIs were narrowed down to signal transduction, ubiquitinization and chemotaxis, all important biological processes related to spermatogenesis (Fig. 4).

Once intra-LRIs were characterised, we determined whether these intra-LRIs also participated in long-range interactions genome-wide in round spermatids, denoting these as inter-chromosomal LRIs (inter-LRIs). This was done by selecting significant higher interactions (z-score interaction >3, see Methods) of intra-LRIs with genomic regions located in other chromosomes (genome-wide approach). As such, we detected the presence of 119 spermatid specific inter-LRIs involving different mouse chromosomes (i.e., multiple interactions) (Fig. 4 and Supplementary Data 2). Out of the total 864 genes contained in inter-LRIs, 71% were protein-coding genes, 12% pseudogenes, 9% ncRNAs and 7% long non-coding RNAs. As for GO terms, we detected enrichment (*p*-value < 0.05 and ES > 1.3, see Methods) for genes associated with negative regulation of peptidase activity (ES = 2.8), G-protein coupled receptor signalling pathway (ES = 2.4), phagocytosis (ES = 2.04), response to cytokines (ES = 1.81), complement activation (ES = 1.68) and arachidonic acid metabolic process (ES = 1.5) (Supplementary Data 3). In contrast to intra-LRI, where 11% (*n* = 77) of the containing genes were DEG, the percentage of DEGs within inter- LRIs increased up to 48.5% (*n* = 419), being genes mainly related with cell surface receptor signalling pathway (ES = 1.66) and translation (ES = 1.39) (Fig. 4).

In terms of structural configuration, intra-LRIs were significantly positively associated with genomic regions that became accessible during meiosis (permutation test based on 10,000 permutations, z-score 78.5, *p* < 0.001) and were located inside TADs (z-score 13.11, *p* < 0.001). Moreover, intra-LRIs were significantly negative associated with CpG islands (z-score −11.9, *p* < 0.001) and meiotic cohesins such as: REC8 (z-score −12.6, *p* < 0.001), RAD21 (z-score −8.8, *p* < 0.001) and CTCF (z-score −3.5, *p* < 0.001). On the other hand, inter-LRIs were not significantly associated with any structural feature.

### Long-range genomic interactions in round spermatids recapitulate ancestral chromosomal configurations

**Fig. 5:**
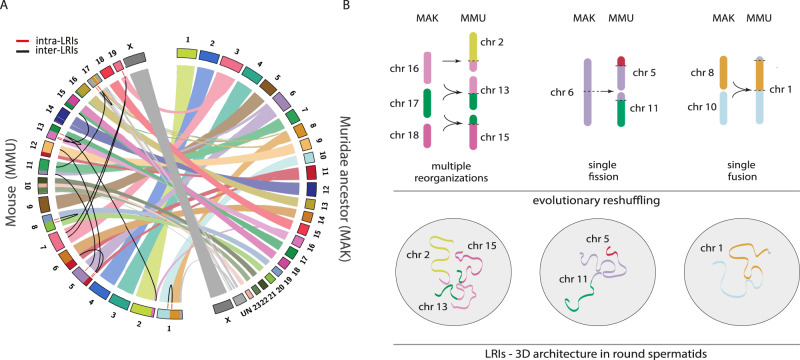
Long-range genomic interactions and ancestral chromosomal configurations. **A** Pairwise circus plot comparing the syntenic regions between the mouse karyotype and the Muridae ancestor karyotype (MAK). Inter-LRIs are depicted in black and intra-LRIs in red. Chromosomes are colour code according to the Muridae ancestor. **B** Upper panel - Schematic representation of the different types of evolutionary rearrangements from the Muridae ancestral karyotype including: single fusions, single fissions and complex reorganisations. Lower panel - Schematic representation of the 3D architecture of LRIs found in round spermatids according to the different types of evolutionary rearrangements. Chromosomes are colour code according to the Muridae ancestor. Abbreviations – LRIs long-ranged interactions, MAK Muridae ancestor karyotype, MMU Mus musculus domesticus, chr chromosome.

To understand the evolutionary implications of LRIs we analysed whether LRIs found in round spermatids were related to the evolutionary history of ancestral chromosomes in rodents. To do so, we analysed the presence of LRIs in the syntenic associations present in recent rodent ancestors, such as Muridae (mouse-rat), Eumuroidea (mouse-rat-Chinese hamster) and Muroidea (mouse-rat-Chinese hamster-mole rat). From the ten syntenic associations found in the Muridae ancestor, seven of them (70%) were now connected by LRIs in mouse spermatids. These included the ancestral syntenies MMU7/19, MMU5/11, MMU17/11, MMU13/15, MMU2/13, MMU5/6, and MMU5/12 (Fig. 5). Similarly, seven syntenic associations (MMU7/19, MMU5/11, MMU13/15, MMU2/13, MMU12/17, MMU5/8 and MMU5/6) from Eumuroidea and 15 syntenic associations (MMU7/19, MMU2/13, MMU11/17, MMU13/15, MMU12/17, MMU1/4, MMU2/4, MMU1/8, MMU5/6, MMU3/5, MMU3/8, MMU17/18, MMU8/15, MMU5/11 and MMU12/14) from Muroidea were found to be connected by LRIs in mouse spermatids (Supplementary Fig 10 and Supplementary Data 4). The percentage of overlap between LRIs and syntenic associations was reduced to 53 and 60% when considering both Eumuroidea (7 out of 13) and Muroidea (15 out of 25) ancestors, respectively suggesting that long-range genomic interactions in round spermatids are associated with recent ancestral chromosomal states (i.e., mouse-rat ancestor).

When analysing into more detail LRIs present in Muridae syntenic associations, we distinguished different types of rearrangements, including single fusions, single fissions and complex reorganisations (Fig. 5). Single fusions from the Muridae ancestor included LRIs in mouse chromosomes 1, 8, and 17, whereas single fissions involved LRIs in ancestral syntenies MMU7/19, MMU5/6 and MMU5/11. On the other hand, LRIs in chromosomes 2, 13 and 15 were involved in complex reorganisations, combining both fusions and fissions. Importantly, EBRs resulting from this evolutionary reshuffling were strongly associated with LRIs (permutation test based on 10,000 permutations, z-score = 136.05, *p* < 0.001). Remarkably, the majority of intra-LRIs (75%) were located at the proximity of EBRs (<2 Mbp), with very few spanning the breakpoint.

Out of 11 fusions-fissions events detected in the Muridae ancestor, 8 were connected by LRIs in mouse round spermatids (11 intra-LRIs and 7 inter-LRIs). These evolutionary LRIs contained a total of 144 genes (30% of them DEG expressed in round spermatids), including 83 (57.6%) protein-coding genes, 35 (24.3%) pseudogenes and 9 (6.3%) ncRNAs. The remaining 6.3% were non-annotated transcripts. As for GO terms, we detected an enrichment (p-value < 0.05 and ES > 1.3) for genes associated with response to pheromone (ES = 8.06), epoxygenase P450 pathway (ES = 7.12) and sensory perception of smell (ES = 2.75), including vomeronasal and olfactory receptors. Additionally, exocrine-gland-secreting peptide family genes (5%)^38^ and *Pramel* genes^39^ were only present in evolutionary LRIs when compared to LRIs not involved in ancestral chromosomal configurations.

## Discussion

Here, we provide evidence for a key role of the 3D chromatin organisation in the formation of transmissible chromosomal reorganisations in the male germline. Using a comprehensive computational approach, we first reconstructed seven ancestors in the rodent lineage with a high degree of accuracy (more than 92% of the mouse genome represented). We then show that EBRs are associated with chromatin environments that become accessible as meiosis progresses, especially in post-meiotic stages of spermatogenesis (i.e., round spermatids), and which are susceptible to DNA damage. Importantly, we also reveal the presence of post-meiotic cell specific long-range interactions, which not only recapitulate evolutionary syntenic associations present in the Muridae ancestor leading to fissions in mouse, but also ancestral chromosome fusions that created new mouse chromosomes.

**Fig. 6:**
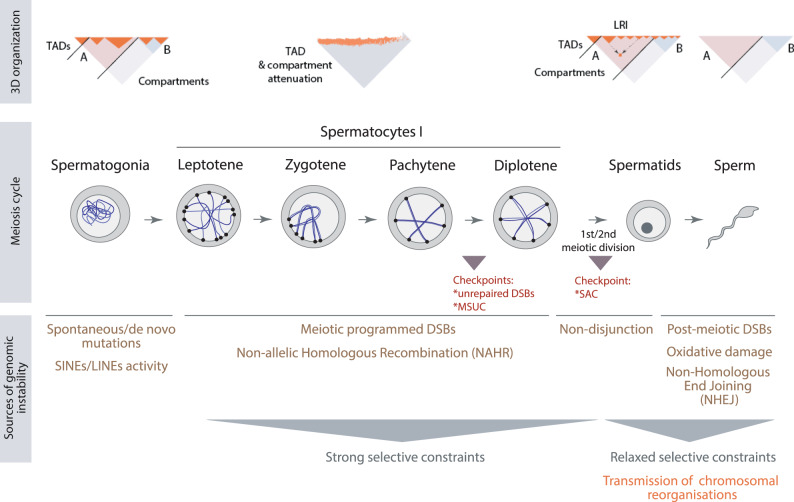
Working model on how chromatin structure of germs cells modulates genome evolutionary plasticity. Upper panel: 3D organisation of meiotic cells. Spermatogonia present a somatic-like organisation with the genome folded into compartments (A and B) and subsequent TADs. Subsequently, there is an attenuation of compartments and TADs in primary spermatocytes (here exemplified as leptotene, zygotene, pachytene and diplotene stages). Post-meiotic cells (round spermatids and spermatozoa) recover a somatic-like configuration, although with particularities such as that TAD borders are not clearly defined, and long-range interactions appear^12,49^. Middle panel: Meiotic progression and checkpoints, Self-renewing spermatogonia commit to meiosis. The primary spermatocytes meiotic prophase I, which is subdivided into four stages: leptotene, zygotene, pachytene and diplotene. The first and second meiotic divisions result in round spermatids, which differentiate into sperm^70^. Meiosis includes three meiotic checkpoints: (i) response to unrepaired double-strand breaks (DSBs), (ii) transcriptional repression called meiotic silencing of unsynapsed chromatin (MSUC), and (iii) the spindle assembly checkpoint (SAC). Lower panel*:* Sources of genomic instability. Spontaneous/de novo mutations and SINEs/LINEs activity predominate in spermatogonia. Meiotic programmed DSBs and its repair through non-allelic homologous recombination (NAHR) are sources of structural genomic changes during prophase I, together with non-disjunction during the subsequent meiotic divisions. The resulting structural changes are under strong selective constrains since errors can be detected by either of the meiotic checkpoints in play. In the case of post-meiotic cells (i.e., sperm) genomic instability can result from oxidative damage and DSBs produced by topoisomerase II DNA disentangling. The repair of DSBs is normally taken place by non-homologous end joining (NHEJ), an error-prone process that can result in structural genomic changes. As no checkpoints are activated in this stage (hence, relaxed selective constrains), there is a high likelihood that chromosomal reorganisations are transmitted to the offspring. Abbreviations – LRIs long-ranged interactions, TADs topological associated domains.

Our results highlight the importance of interpreting the dynamic spatial genome organisation of germ cells in the context of germline DNA damage responses (DDR) and meiotic checkpoints (Fig. 6). During gametogenesis, there are two key waves of DNA damage induction that may trigger genomic rearrangements: (i) programmed meiotic DSBs catalysed by SPO11 in early stages of male and female prophase I^21^, and (ii) male-specific induction of DSBs in elongating spermatids to relieve torsional stress during chromatin condensation^40^. Here we used the genomic distribution of DMC1 together with PRDM9 binding sites as a proxy for meiotic DSB locations (SPO11-oligos hotspots^41^). As both male and female meiotic DSBs are mainly driven by the same mechanism (despite differential efficiencies^42^), the DMC1 and PRDM9 data analysed, though generated in males, encompass the majority of female meiotic DSB hotspots. Importantly, we detected that chromosome rearrangements were not associated to meiotic DSBs and did not disturb meiotic chromosomal architecture in prophase I. Although it was previously shown that both SPO11 hotspots and H3K4me3 marks positively correlated with cohesin occupancy^12^, A compartments^12^ and open chromatin states (this study) in primary spermatocytes, EBRs (irrespective of the rodent ancestor considered) were significantly devoid of DMC1 and PRDM9 sites. Conversely, EBRs were very strongly associated with the location of DSB hotpots in post-meiotic spermatids, consistent with the fact that such DSBs must be repaired via a less accurate mechanism such as NHEJ or MMEJ since no sister chromatid is present^23,37^. We therefore conclude that transmissible rearrangements are more strongly associated with male specific post-meiotic DNA damage locations than with non sex specific meiotic DSB locations.

The permissiveness of some genomic regions to undergo chromosomal breakage can also be associated with changes in chromatin accessibility^3,10,43^. This has been recently demonstrated in somatic cells, where TAD boundaries experience strengthening upon induced DSBs to accommodate accessibility of the plethora of proteins involved in the DDR^44^. Likewise, it has been shown that highly interacting regions are normally transcriptionally active^45^ and that promoter-enhancer interactions can be facilitated by chromatin remodelling^46^. This view aligns with the growing evidence pointing to an association between EBRs and TAD boundaries in mammalian and bird somatic cells^3,10,47,48^. However, this poses a paradox in that rearrangements that occur at TAD boundaries are selectively favoured because they are less likely to disrupt gene regulation, but the DNA strand breaks that initiate rearrangements are more likely to occur at open chromatin within TADs. Here, we resolve this paradox by showing that EBRs are selectively associated with regions that form ‘closed’ chromatin in pre-meiotic (spermatogonia) or meiotic (primary spermatocytes) cell types and only become ‘open’ chromatin in spermatids. In particular, these regions lie within TADs in spermatids but are near TAD boundaries in other cell types (Fig. 6).

Crucially, our analysis of contact frequency maps during spermatogenesis detected the presence of cell-specific long-range interactions that can recapitulate ancestral chromosomal states. We have previously shown that chromosomal reorganisations have an impact on 3D genome topology of germ cells in two ways: (i) altering chromosomal nuclear occupancy, and (i) reshaping landscapes of recombination^16,49^. Importantly, the redistribution of chromosomal nuclear occupancy in spermatocytes that result from chromosomal fusions brings new genomic regions into close proximity, predisposing to the occurrence of additional rearrangements and expose chromosomal domains to novel regulatory environments, potentially affecting gene expression and/or regulation^16^. Here we demonstrate that this can be the case, as reflected by the presence of cell-specific long-range genomic interactions in mouse round spermatids containing genes relevant for spermatogenesis, fertilisation and sperm chemotaxis. Critically, relevant inter-chromosomal long-range interactions now present in mouse round spermatids correspond to genomic regions that were present in single chromosomes from the recent Muridae ancestor; while current intra-chromosomal long-range interactions in the present day mouse round spermatids correspond to regions that were present in two or more chromosomes in the Muridae ancestor.

We therefore propose that the landscape of chromosomal reorganisations (fusions and fissions) that took place during genome evolution in rodents is linked to the chromatin context now present in mouse round spermatids. When reorganisations occur within 3D contact hubs, this may either separate interacting regions (converting intra- to inter-chromosomal LRIs) or conversely bring them together. Alternatively, when chromosomal fissions occur, new inter-chromosomal LRIs may be created in order to maintain critical associations between regions that were formerly contiguous. This is in fact a crucial aspect that links the dynamic spatial genome organisation of germ cells with evolutionary chromosomal reorganisations.

Our model also integrates the role of meiotic checkpoints in genome evolutionary plasticity. Once a rearrangement has occurred by breaking and rejoining of the DNA, it must evade elimination by cell cycle checkpoints, and/or viability selection on the resulting offspring. Germ cells have a complex surveillance network including three major meiotic checkpoints: (i) response to unrepaired DSBs, (ii) transcriptional repression called meiotic silencing of unsynapsed chromatin (MSUC), and (iii) the spindle assembly checkpoint (SAC)^31^. Therefore, any chromosomal rearrangement occurring before or during meiosis has a high probability of leading to meiotic arrest due to the activation of any of these three checkpoints (Fig. 6). Moreover, for inter-chromosomal translocations, even in the event that such a rearrangement occurs in pre-meiotic cells and is not eliminated by the meiotic checkpoints, around 50% of the resulting gametes will be aneuploid and unlikely to lead to viable offspring. Thus, the vast majority of rearrangements occurring before or during meiosis will be eliminated. This view aligns with our observation of EBRs being devoid of programmed meiotic DSBs and meiotic cohesins in primary spermatocytes. Conversely, genomic rearrangements occurring in post-meiotic cells are not subject to meiotic checkpoints and are moreover guaranteed to be euploid since each cell contains exactly one haploid genome. Therefore, chromosomal reorganisations occurring after meiosis have a higher probability to be transmitted than genetic insults occurring before or during meiosis (Fig. 6). And this is in fact what we observe in our computational approach, as EBRs are strongly associated with genomic regions of DNA damage in round spermatids.

Summarising, our observations suggest that chromatin remodelling during spermatogenesis represents an emerging framework where evolutionary genomic variation can be generated and transmitted to the offspring. Understanding how genome instability affects gene expression and regulation in the germ line will allow to further determine the effect of genome reshuffling on evolution and reproduction.

## Methods

### Ancestral karyotype reconstruction

The genome assemblies of 14 Rodentia species representatives of the major phylogroups, assembled at chromosome-level or with scaffold N50 > 3 Mbp (Supplementary Table 1), and two mammalian outgroup species (human and rabbit) were used to generate pairwise alignments with the mouse genome (mm10) using LASTZ with default parameters. LASTZ alignments were converted into chain and net files using Kent toolbox utilities using the parameters -minScore = 1000, -linearGap = medium, C = 0, E = 30, K = 3000, L = 3000, O = 400. The Y chromosome was omitted due to the difficulty in assembling it to a sufficient degree of quality, enrichment of repeats and palindromes in the chromosome^50^. The coverage of nets of each species was calculated against mm10 to minimise the potential fragmentation introduced into the reconstruction of the ancestral karyotypes.

Reconstructed ancestral chromosome fragments (RACFs) were generated by DESCHRAMBLER algorithm^28^ using a syntenic fragment resolution of 300Kbp and a minimum adjacency score of 0.0001. Pairwise divergence times between the house mouse genome and each of the studied species were found using TimeTree^51^. The divergence times between species were then used to write a phylogenetic tree in Newick format and visualised using FigTree^52^. We reconstructed seven different ancestors in the rodent lineage: Muridae, Eumuroidea, Muroidea, Myodonta, the ancestor for the mouse-related lineage, the ancestor of all rodents except squirrels, and the Rodentia ancestor.

The number of RACFs produced from DESCHRAMBLER was higher than the number of chromosomes suggested by previous studies^53,54^. Consequently, the adjacent RACFs in each of the reconstructed ancestors were manually merged using both reference genome and other reconstructed ancestors, which were most closely related. This process was started on the Muridae ancestor using the house mouse genome as a point of reference, before working back in evolutionary time using the closest related ancestors as a point of reference. To determine the final number of ancestral chromosomes, we followed the model suggesting that there is a conserved boundary of chromosome-size variation in all mammals^29^. Average chromosome length was calculated for all genomes assembled at chromosome level and minimum size boundary was estimated to be 26 Mbp. RACFs smaller than this boundary were labelled as unplaced. Plots of the RACFs were produced with the R package syntenyPlotteR^4^.

### Detection of Evolutionary Breakpoint Regions (EBRs)

The evolutionary breakpoint regions (EBRs) in each lineage were identified by considering the mouse genome as a reference. EBRs were counted from the coordinates of manually merged RACFs. The minimum size shared across all lineages was considered as a breakpoint. The EBRs were phylogenetically classified depending on the ancestral lineage in which they occurred. EBRs were further separated by the type of rearrangement they delimited into inversion EBRs or inter-chromosome EBRs, if they demarcated inversions or were the result of fusion or fission events, respectively.

### Histone, ATAC and DSBs profiles

A total of 27 fastq files were downloaded from NCBI (Supplementary Table 3). Read quality was checked using FastQC (v0.11.9)^55^. Reads were trimmed using Trimmomatic (v0.39)^56^, with SE/PE setting used depending on the read type. Adapter sequences were removed using ILLUMINACLIP, as well as reads with an average Phred score of <30 or <20 with the AVGQUAL:20 or 30 for DSB and other libraries, respectively. MINLEN:30 was also used for DSBs libraries. Trimmed fastq files were aligned to the mouse genome mm10 using bwa-mem (v0.7.17-r1188)^57^. Samtools merge was used to combine BAM files of the same histone mark.

Histone mark data was analysed using MACS2 (v.2.2.7.1)^58^ with either the default settings of callpeak to produce narrow peaks or with–broad–broad-cut-off 0.05 to produce broad peaks. Histone marks were defined as narrow or broad based on the ENCODE project^59^. For marks not defined on ENCODE, the broad settings were used. ATAC-seq data was analysed using MACS2 (v.2.2.7.1)^58^ with the default settings of callpeak to produce narrow peaks. As for DSBs, data was analysed using the same parameters described in the original study^24^. Briefly, MACS2 with the default settings and–bw600 -q0.01–broad–broad-cutoff 0.1 were used. MACS2 files of the different round spermatid stages (stage 1–9 and stage 15–16) were merged for further analysis to facilitate cross-comparison with datasets not stratified into different developmental stages in spermatids. Longest DSBs peaks were visualised in IGV (v2.12)^60^ and co-locate with large stretches of alpha satellite regions. Because alpha satellites are not fully assembled in the centromeres of all mouse chromosomes, to avoid bias towards the assembled stretches of satellite regions, we excluded these DSBs peaks from further analysis.

ChromHMM (v1.22) was used for chromatin state analysis applying the default binsize of 200 bp with the concatenated strategy^61^. The corresponding cell type specific input was used as a control to adjust the binarization threshold locally. Once binarization was completed the model was learned with varying numbers of states, with 8 states (from E1 to E8) chosen as the optimum model.

To identify how chromatin states change during spermatogenesis, genomic locations of states in each cell type (spermatogonia, spermatocytes and spermatids) were compared using bedtools intersect^62^. Regions of the genome missing a chromHMM state in any cell type were removed. The dominant states in any of the three cell types (E7, E3 and E1) were merged into a joint state named E0. Genomic locations were then labelled according to the states in each cell type, and the transitions from one chromatin sate to another were plotted using ggalluvial with ggplot2 in R. Consecutive 200 bp regions with the same three-cell type state combination were merged, and 34 combinations with more than 0.1% genomic coverage were identified. This cut off was chosen because the total coverage of all these regions represented >98% of the mm10 genome.

### Hi-C data

Hi-C data from mouse spermatogonia, primary spermatocytes and round spermatids was obtained from GEO:GSE132054^12^ and processed with TADbit (v0.2.0.23)^63^ and HiCExplorer (v3.6)^12,64^. Contact matrices were built at 50 kb resolution and normalised to 100 M reads.

### Long-range interactions

Hi-C interactions matrices at 50 Kbp resolution were exported to Ginteractions format using HiCExplorer (v3.6). Intra-chromosomal Hi-C interactions matrices were used as input to define long-range interacting (LRI) regions for each mouse chromosome. This was done by selecting 50 Kbp bins with interactions higher than 100 that occurred at bins separated by at least 10 Mbp. Out of the resulted bins false positives were eliminated by removing low mappability regions. The mappability of each region was calculated using GenMap (v1.3)^65^, removing regions with mappability below 0.5. Inter-chromosomal long-range interactions were identified based on a z-score > 3.

To evaluate whether intra-chromosomal LRI regions significantly interact more with other regions in the genome, we created 1000 sets of regions with the same length and distribution of the intra-chromosomal LRI regions, using bedtools shuffle v2.29.2^62^. Genomic regions contained in the random 1000 sets were extracted automatically using and in-house R script from the 50 kb inter-chromosomal Hi-C interactions spermatids matrix. The interaction value of all these regions was then transformed into Z-score to address the significant differences.

### Gene ontology enrichment

Using BioMart v2.46.1 in R v4.0.3, unique protein coding gene IDs in mm10 for each classification were identified. These were input into the PANTHER db^66^ for the gene ontology (GO) enrichment. Statistical overrepresentation test was selected with either GO biological process complete or PANTHER (v17.0) pathways. Only GO terms with ≥1.5-fold enrichment and FDR < 0.5 were considered statistically significant. Plots were created with ggplot2 v3.3.5 in R. In the case of LRIs, where non-coding transcripts were detected, DAVID (v6.9) was used for the GO enrichment considering an enrichment cluster score (ES) > 1.3 as default parameter^67^.

### Differentially expressed genes

RNA-seq data from mouse spermatogonia, primary spermatocytes and round spermatids was obtained from GEO:GSE132054 and processed using AIR (https://transcriptomics.sequentiabiotech.com/)^12^. The resulting expression file was then analysed following the manual of the R package for empirical analysis of gene expression data: edgeR (v3.13)^68^. Then, the edgeR algorithm glmQLFTest with default parameters (*p*-value < 0.05; log2FoldChange = ± 1) was used to determine the differentially expressed genes, using spermatids as a reference.

### Multi-association analyses

Statistical association between different genomic features was evaluated using the RegioneR R package version. 1.26^69^. Based on the regioneR package we have created a series of functions to allow the calculation of associations between multiple regionsets. Due to the implementation of multiple comparisons, the *p*-value calculation was adjusted using Benjamini–Hochberg procedure. The value of the z-score for association with an adjusted *p*-value greater than 0.005, was considered as 0. The z-score calculated was subsequently normalised by dividing it by the square root of n where n is the number of regions present in the permuted regionset. All the permutations were performed using randomizeRegions and NumOverlaps respectively as randomisation and evaluation function. The genomic positions of gene, LINEs, LTRs, and ERVs for mm10 genome were downloaded from the UCSC browser and used as input for our analysis.

### Quantification and statistical analysis

The statistical analyses were performed using R. Statistical parameters and tests are reported in the Figures and Figure Legends when necessary.

### Reporting summary

Further information on research design is available in the Nature Research Reporting Summary linked to this article.

## Supplementary information

Supplementary Information

Description of additional Supplementary Files

Supplementary Data 1

Supplementary Data 2

Supplementary Data 3

Supplementary Data 4

Reporting Summary

## Data Availability

The data that support this study are available from the corresponding authors upon reasonable request. The Hi-C, RNA-seq and cohesion ChIP-seq datasets from mice used in this study were retrieved from the NCBI GEO repository: GSE132054 (Spermatogonia Hi-C: GSM3840080; Spermatocytes I Hi-C: GSM3840082 and Spermatids Hi-C: GSM3840083; Spermatogonia RNA-seq: GSM3840094; Spermatocytes I RNA-seq: GSM3840095; Spermatids RNA-seq: GSM3840096; Spermatocytes I CTCF, RAD21L, REC8: GSM3840086, GSM3840087, GSM3840088 and Spermatids CTCF, RAD21L, REC8: GSM3840089, GSM3840090, GSM3840091). DSBs data from mice used in this study were retrieved from the ENA repository under the accession code PRJEB20038. ChIP-Seq data for H3K4me3, H3K27me3 and H3K27ac modifications from mice was retrieved from the NCBI GEO repository: GSE49624 (Spermatogonia H3K4me3, H3K27me3 and H3K27ac: GSM1202705, GSM1202708, GSM1202713; Spermatocytes I H3K4me3, H3K27me3 and H3K27ac: GSM1202706, GSM1202709, GSM1202714; Spermatids H3K4me3, H3K27me3 and H3K27ac: GSM1202707, GSM1202710, GSM1202715; Spermatogonia input DNA: GSM1202723; Spermatocytes I input DNA: GSM1202724 and Spermatids input DNA: GSM1202725). ATAC-seq data were retrieved from the NCBI GEO repository: GSE102954 (Spermatocytes I replicate 1 and 2: GSM2751129, GSM2751130 and Spermatids replicate 1 and 2: GSM2751133, GSM2751134). Source data are provided with this paper.

## Code availability

All functions for the multi-association analyses can be found at https://github.com/RMalinverni/GenoMatriXeR/ (v1.0.0, 10.5281/zenodo.6379726). The rest of functions used can be found at https://github.com/Farre-lab/EBRs_HiC_Spermatogenesis_paper (v1, 10.5281/zenodo.6376838).
