# Supplementary material for: 3D chromatin remodelling in the germ line modulates genome evolutionary plasticity
Source: Nat Commun. 2022 May 11;13:2608. doi: 10.1038/s41467-022-30296-6 (PMC9095871; doi:10.1038/s41467-022-30296-6)
Supplement: Supplementary file 1 — Supplementary Information [file 41467_2022_30296_MOESM1_ESM.pdf]

Supplementary Information

3D chromatin remodelling in the germ line modulates genome evolutionary plasticity

Álvarez-González et al.

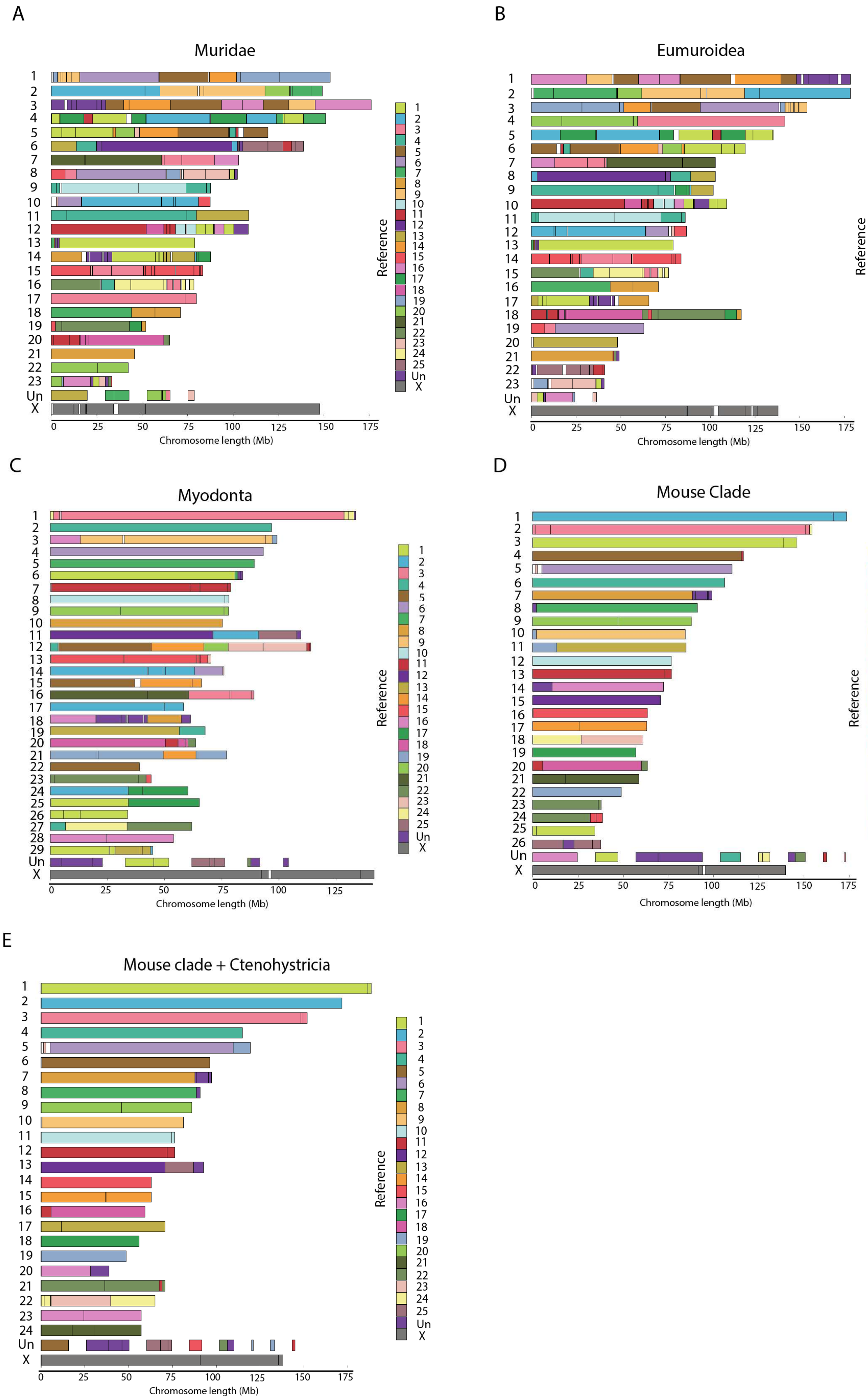

Supplementary Figure 1: **Ancestral karyotypes.** Reconstructed ancestral karyotypes for the ancestors of (A) Muridae, (B) Eumoroidea, (C) Myodonta, (D) mouse clade and (E) mouse clade + Ctenohystrica. Ancestral karyotypes are coloured according to Rodentia RACFs. Related to Figure 1.

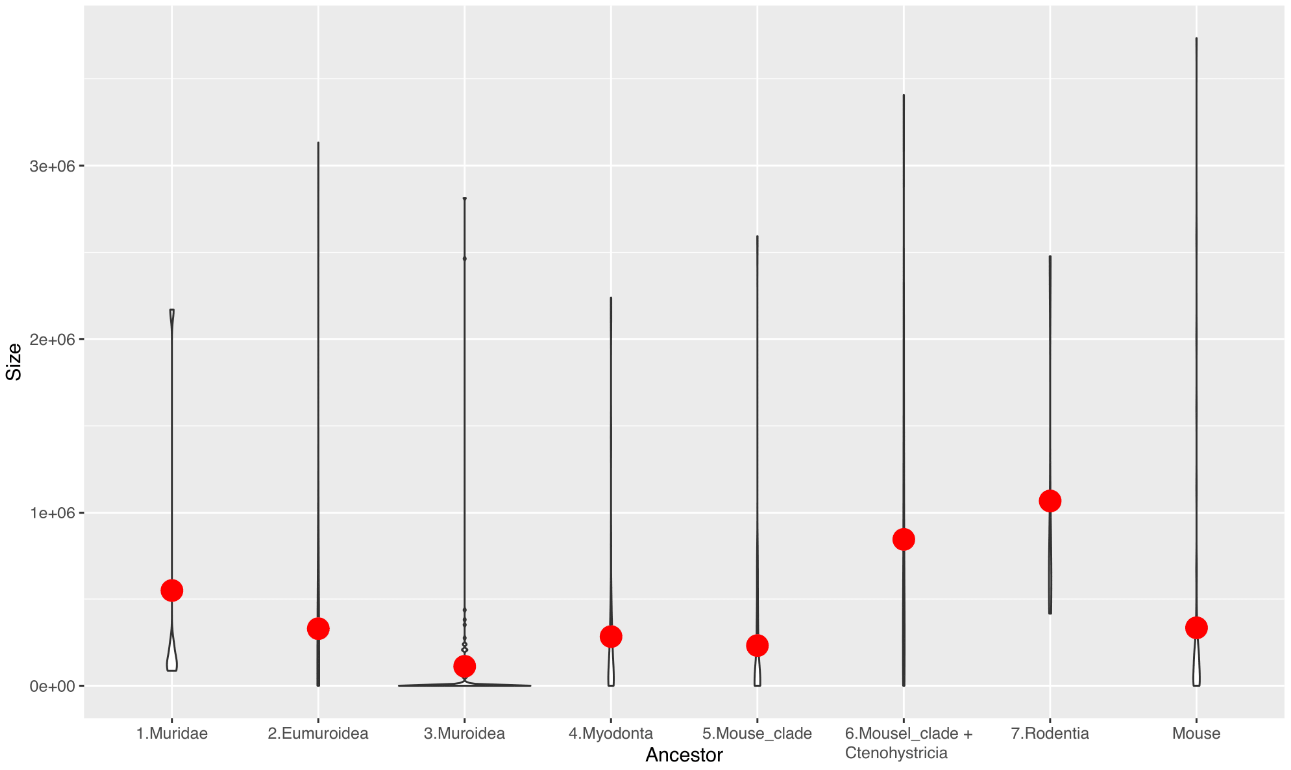

Supplementary Figure 2: **EBRs genomic size**. Variation in genomic size (in Mbp) of EBRs detected in each ancestor: Muridae (n=5), Eumoroidea (n=13), Muroidea (n=75), Myodonta (n=41), the mouse clade (n=30), the mouse clade + Ctenohystrica (n=10), Rodentia (n=4) and mouse (n=54). Red circles denote EBRs median sizes. Related to Figure 1.

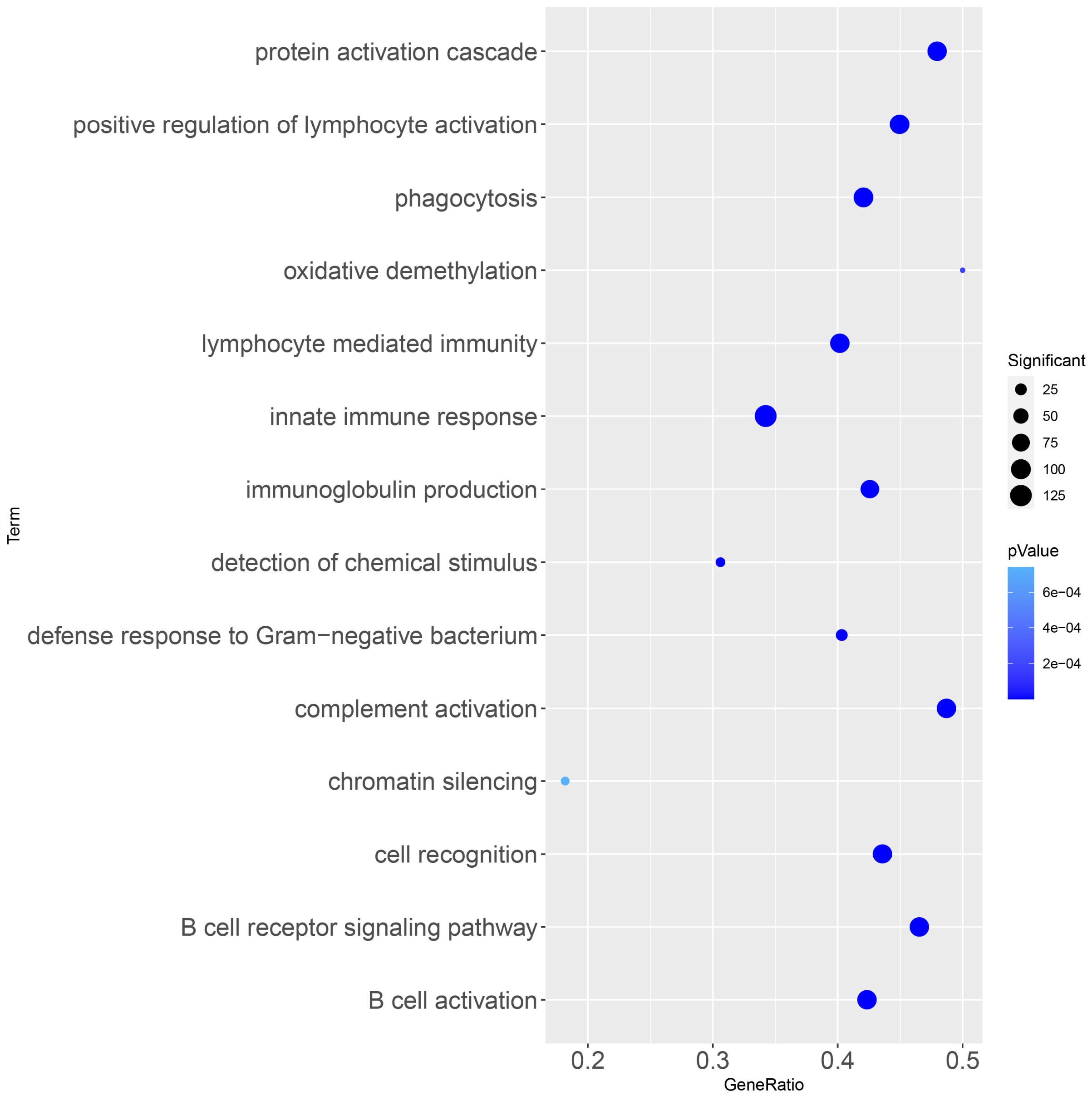

Supplementary Figure 3: Gene Ontology (GO) terms enriched in EBRs. Bubble size depicts the number of genes annotated in each GO term. Bubble shade represents the p value with darker shades for lower p values. The raw p-values are determined by Fisher's exact test and the False Discovery Rate as calculated by the Benjamini-Hochberg procedure. The x-axis shows the ratio of genes annotated for each GO term in the analysed list versus the background list. Related to Figure 1.

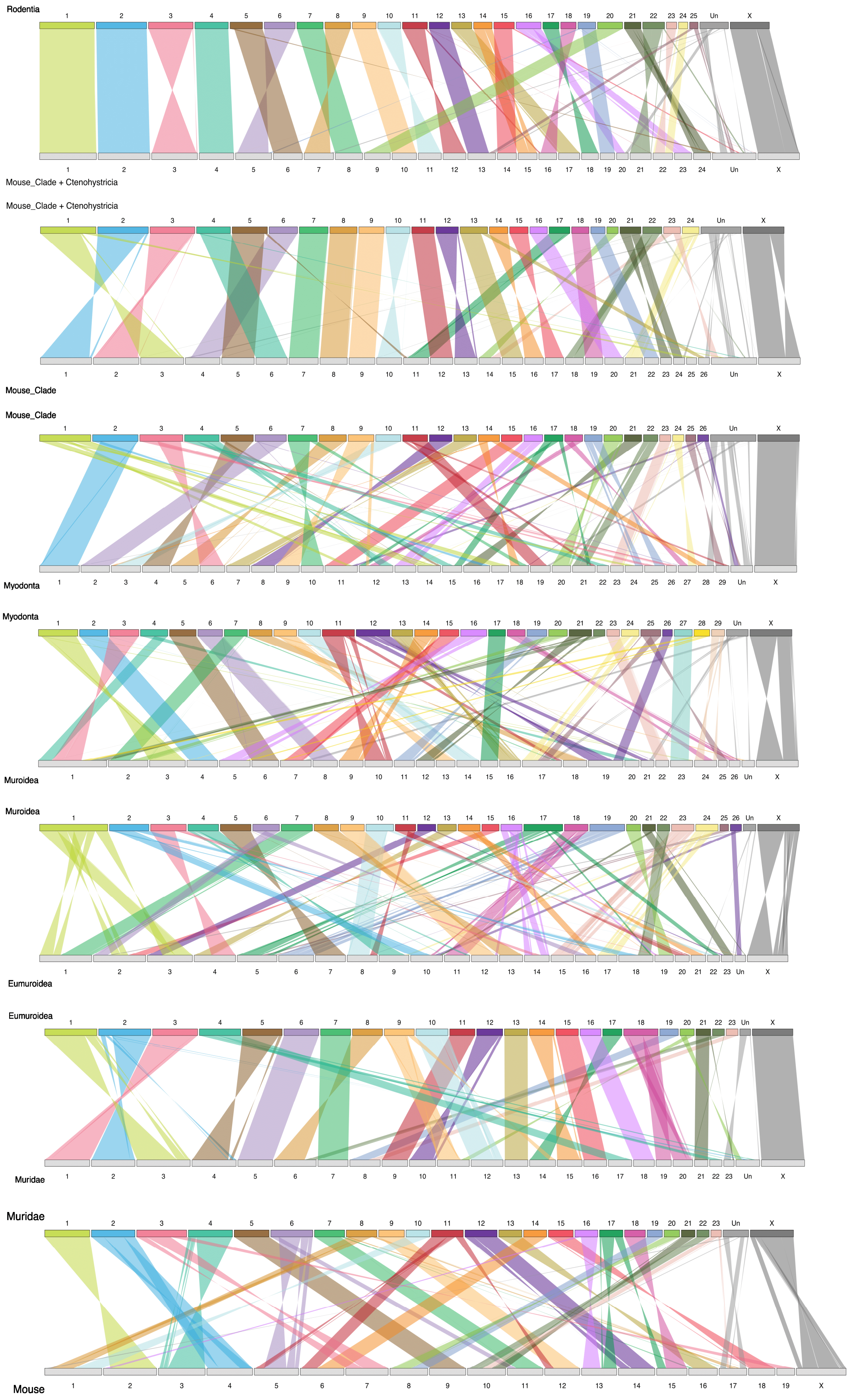

Supplementary Figure 4: **Evolutionary reshuffling between the Mouse clade ancestor and Myodonta.** Each ribbon represents the syntenic fragments between the ancestors, whereas tilted ribbons indicate inversions. Syntenic fragments are coloured according to Rodentia RACFs. Un: unplaced scaffolds. Related to Figure 1.

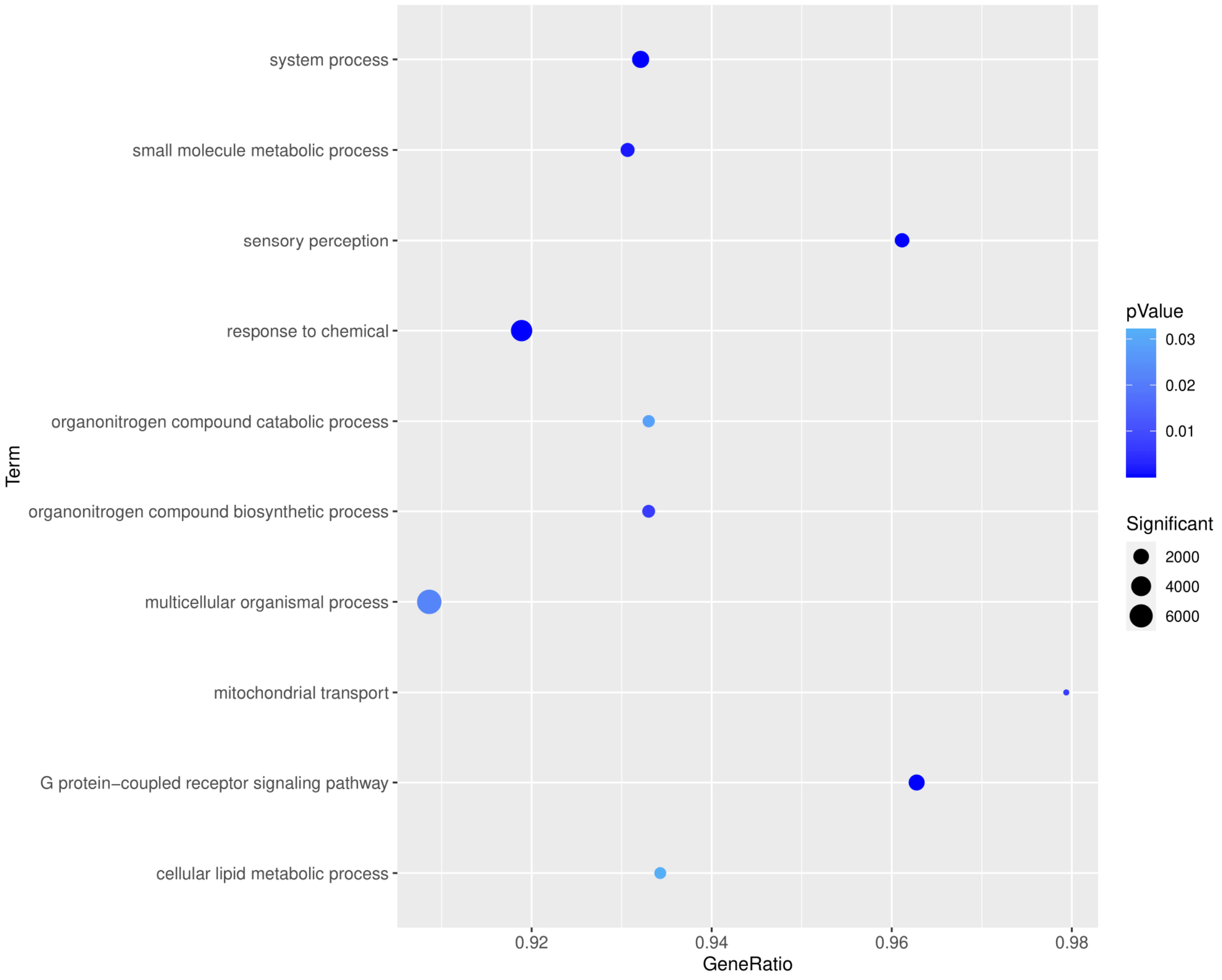

Supplementary Figure 5: **Gene Ontology (GO) terms enriched in msHSBs.** Bubble size depicts the number of genes annotated in each GO term. Bubble shade represents the p value with darker shades for lower p values. The raw p-values are determined by Fisher's exact test and the False Discovery Rate as calculated by the Benjamini-Hochberg procedure The x-axis shows the ratio of genes annotated for each GO term in the analysed list versus the background list. Related to Figure 1.

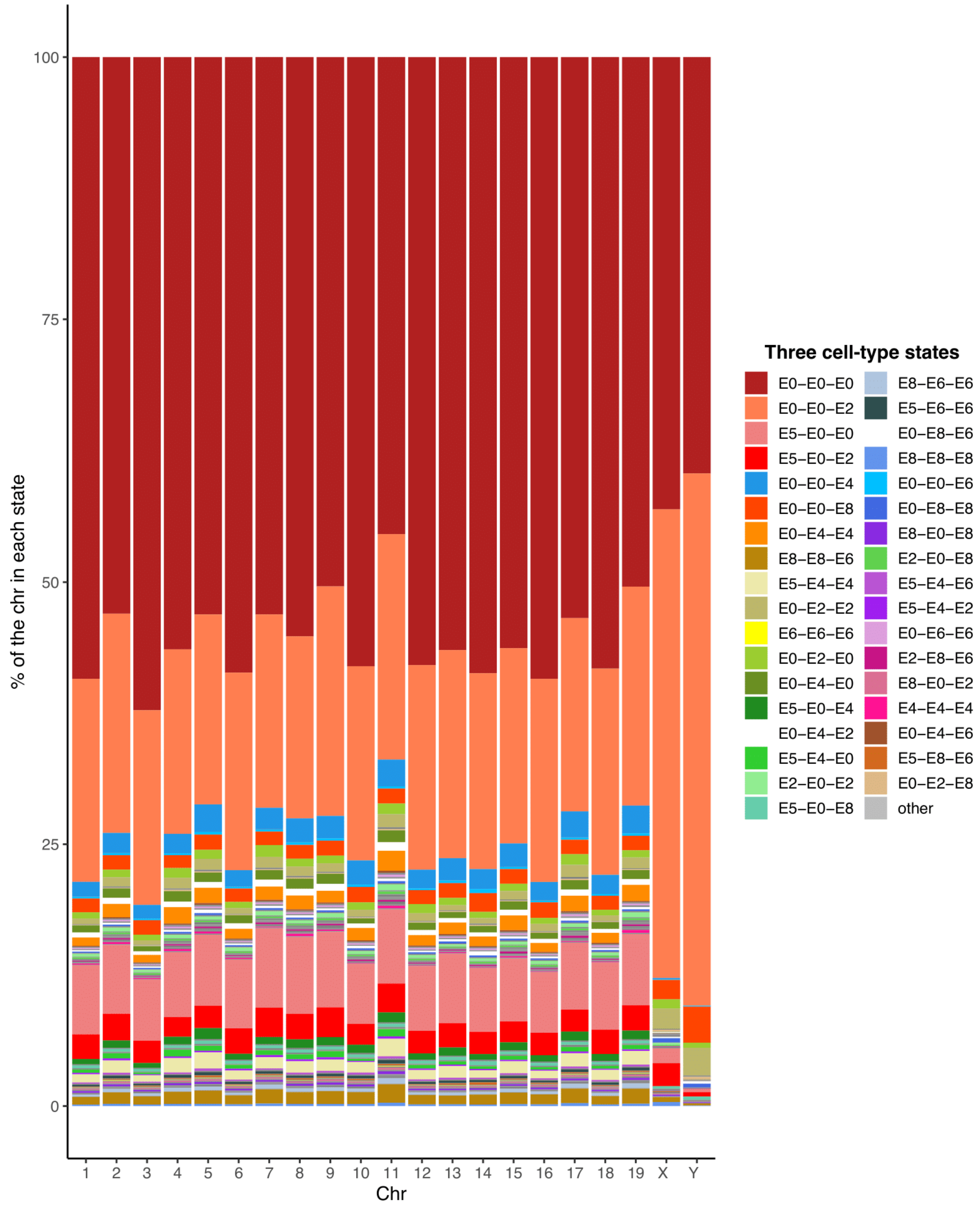

Supplementary Figure 6: **Genomic coverage of chromatin state transitions.** Percentage of coverage found per mouse chromosome of the 35 most frequent chromatin state transitions from spermatogonia, primary spermatocytes and sperm. Related to Figure 2.

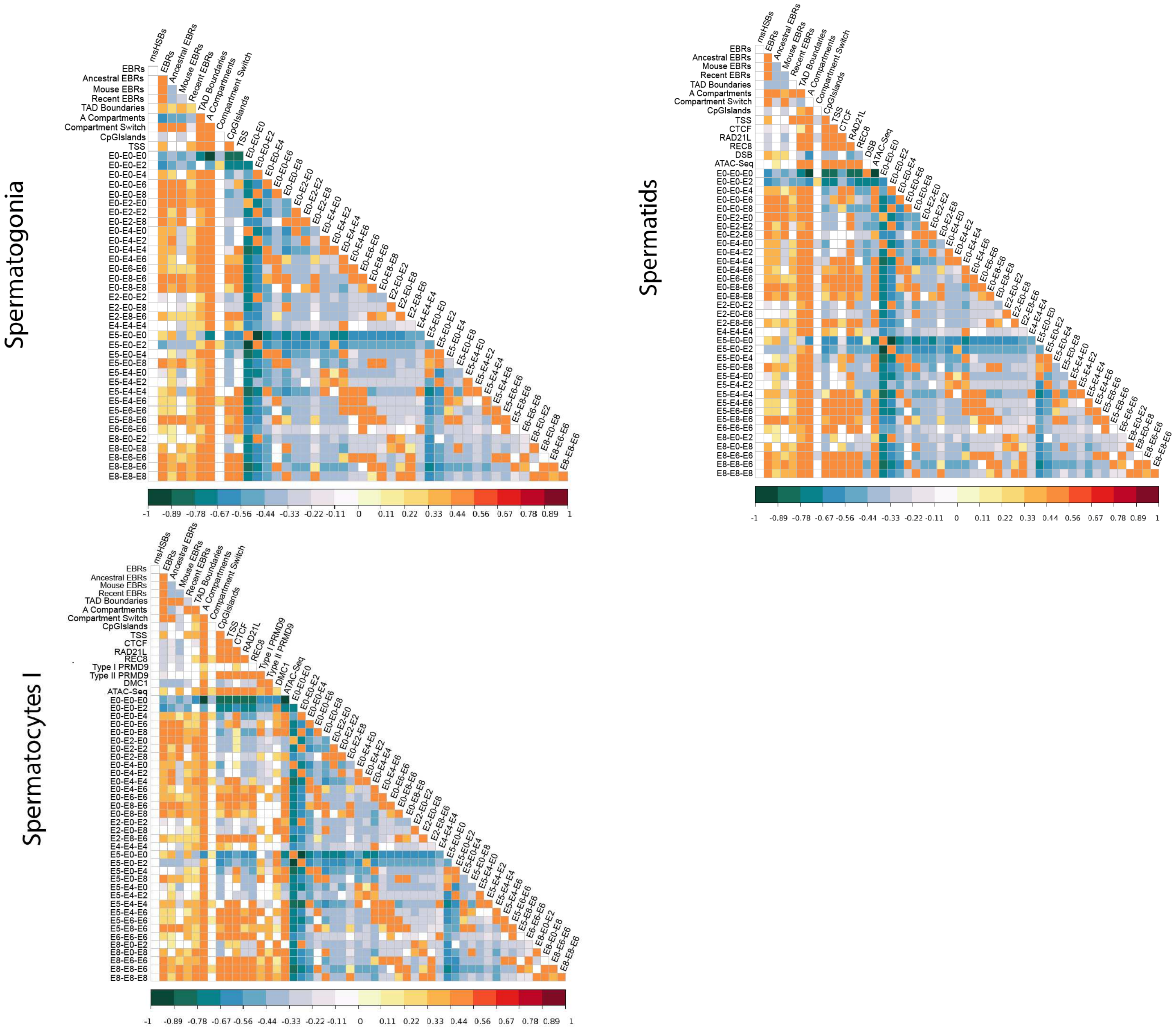

Supplementary Figure 7: Multi-comparison analysis. Heatmaps displaying correlations between different genomic features obtained by regioneR (multicomparison) in all three cell types: spermatogonia, primary spermatocytes and round spermatids. Genomic features included: mHSBs, EBRs (ancestral, recent and mouse specific), TAD boundaries, A compartments, compartment switch (from A to B and viceversa), CpG islands, transcription start sites (TSS) and chromatic transitions between chromatin states (E) in the three cell types. CTCF, cohesins (RAD21L and REC8) and ATAC-seq were included for both primary spermatocytes and round spermatids. Primary spermatocytes also included PRDM9 sites (Type I and II) and DMC1 sites. Round spermatids included post-meiotic DSBs (DSB). Related to Figure 3.

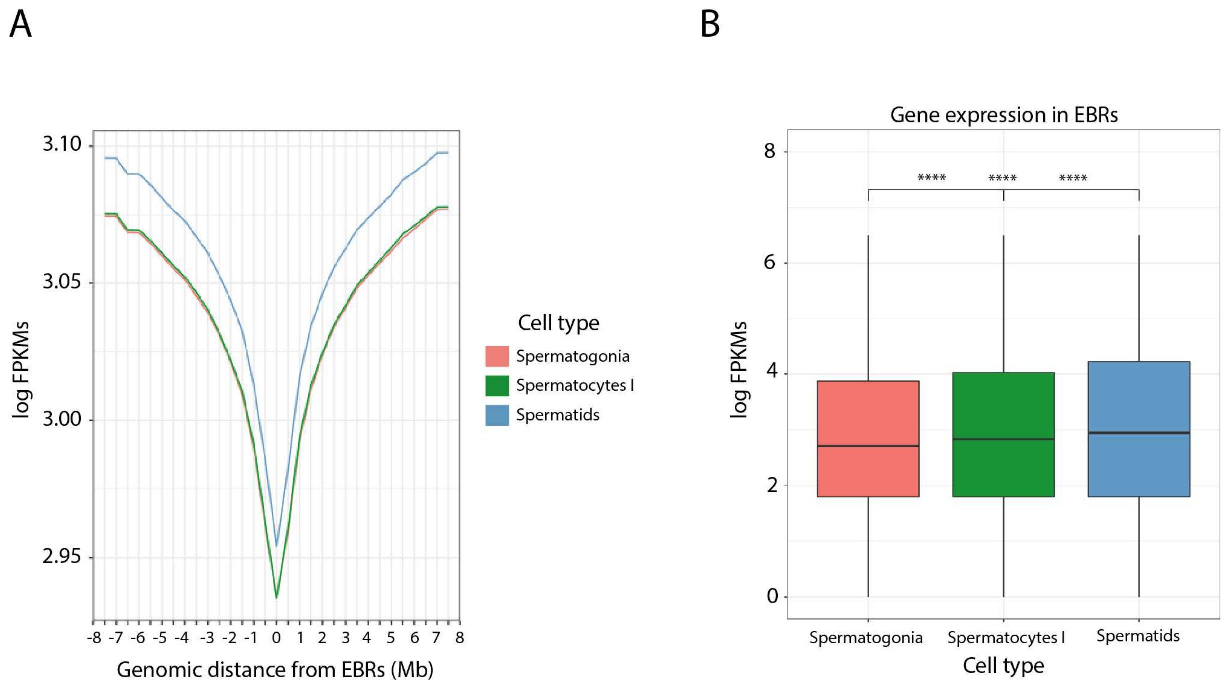

Supplementary Figure 8: **Expression levels at EBRs in mouse germ cells.** (A) Expression levels (represented as log FPKM values) of transcripts located at EBRs in all three cell types (spermatogonia, primary spermatocytes and round spermatids). (B) Boxplots representing mean values of expression levels (as log FPKM values) of EBRs (n=232) in spermatogonia, primary spermatocytes and round spermatids. Boxplots are presented as mean values (center line) ± SD. Asterisks represent statistically significant differential gene expression between cell types (Mann-Whitney test, ****p<2.2e-16, two-sided). Related to Figure 3.

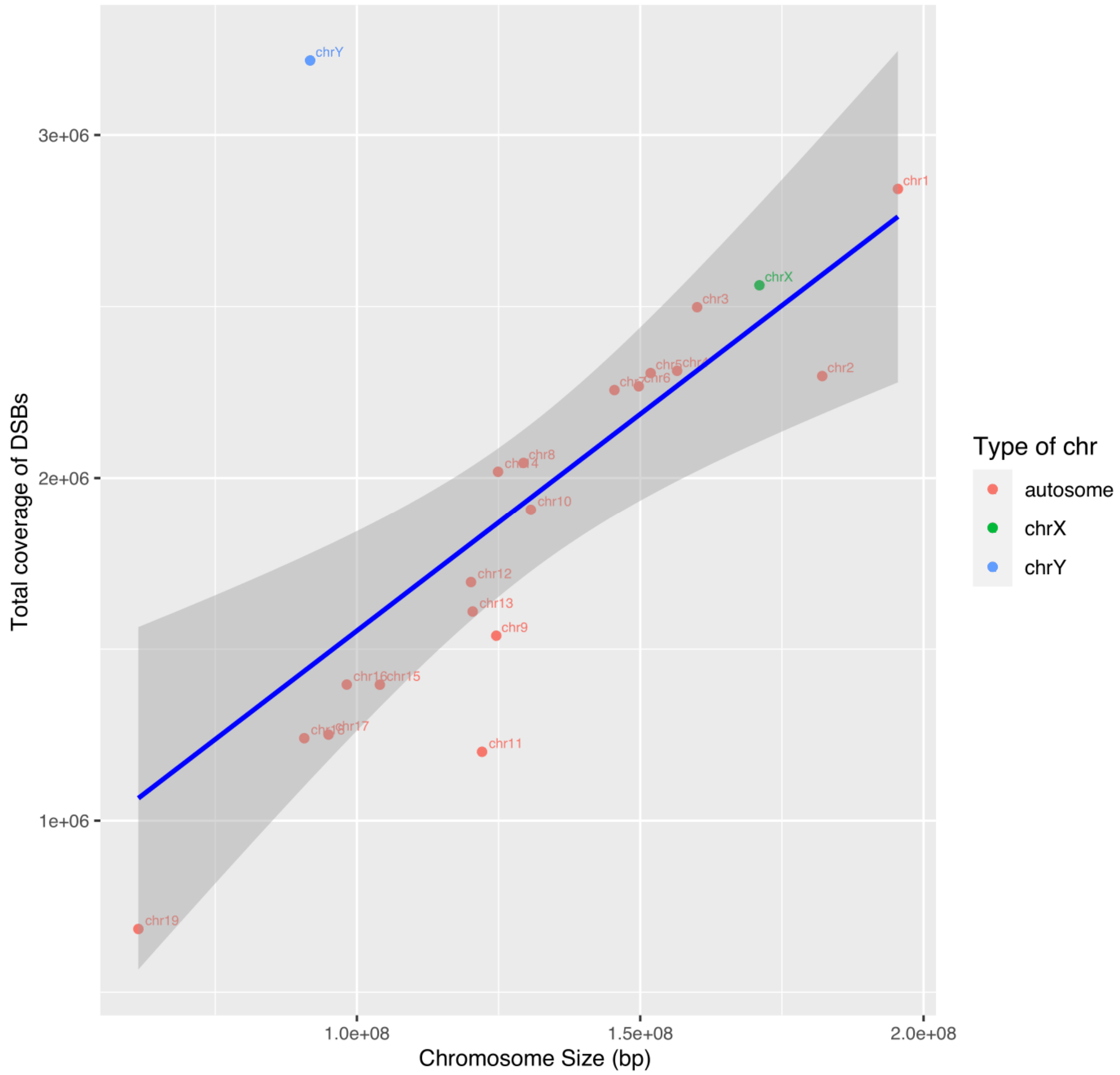

Supplementary Figure 9: **Correlation between post-meiotic DSBs and chromosomal size.** Linear regression of the number of post-meiotic DSBs (expressed as total bp coverage) detected in mouse chromosomes. Autosomes are depicted in red, the X chromosome in green and the Y chromosome in blue. Grey shading represents 95% confidence interval. Related to Figure 3.

A

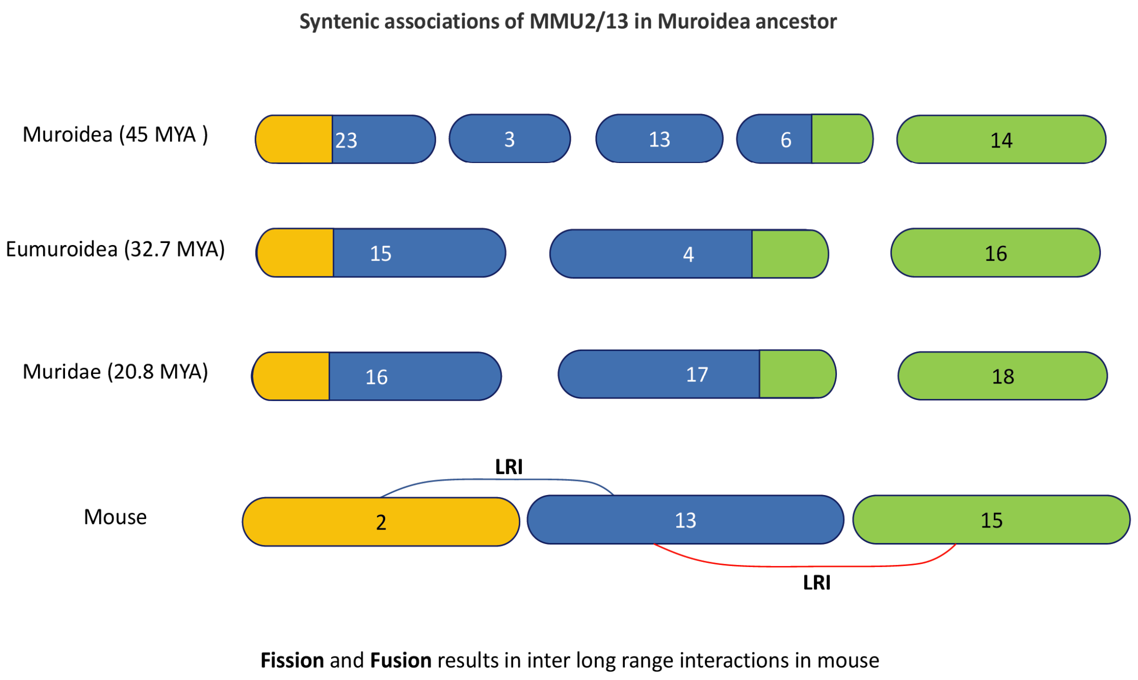

B

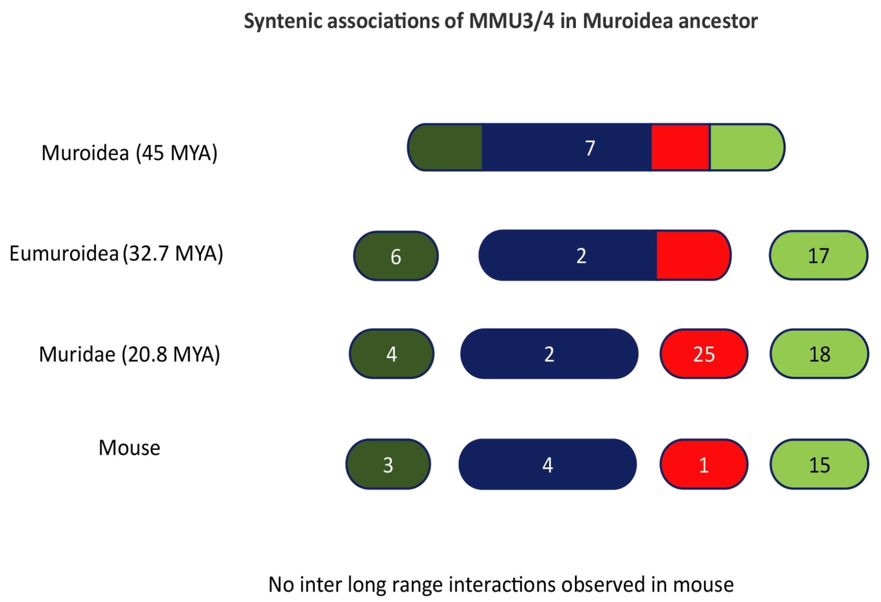

Supplementary Figure 10: Long-range genomic interactions and ancestral chromosomal configurations. (A) Syntenic association of mouse chromosomes 2 and 13 in Muroidea, Eumuroidea and Muridae ancestors showing that LRIs recapitulate recent ancestral syntenic associations. (B) Syntenic association of mouse chromosomes 3 and 4. MMU3/4 was one single chromosome in Muroidea ancestor, then a fission into two or more chromosomes happened in recent ancestors. In this case, no LRIs were detected in mouse. Rectangles represent chromosomes, while lines connecting them are LRIs. Related to Figure 5.

Organism	Diploid no. (2n)	Family	Genome size (Gbp)	No. scaffolds	Scaffold N50 (Mbp)	Genome version
*Mus musculus**	40	Muridae	2.8	N/A	-	GRCm38.p6
*Rattus norvegicus**	42	Muridae	2.9	N/A	-	Rnor_6.0
Cricetulus griseus	22	Cricetidae	2.6	8.264	62	CriGri-PICR_HiC
Microtus ochrogaster	54	Cricetidae	2.3	N/A	-	MicOch1.0
Nannospalax galili	-	Spalacidae	3.1	154.976	3.6	S.galili_v1.0
Jaculus jaculus	48	Dipodidae	2.9	10.898	22.1	JacJac1.0
Dipodomys ordii	72	Heteromyidae	2.2	65.193	11.9	Dord_2.0
Castor canadensis	40	Castoridae	2.5	6.496	136.7	C.can_genome_v1_HiC
Ictidomys tridecemlineatus	34	Sciuridae	2.5	12.483	8.1	SpeTri2.0
Marmota flaviventris	42	Sciuridae	2.6	32.915	17.2	ASM367607v1
Fukomys damarensis	-	Bathyergidae	2.3	73.969	62.6	DMR_v1.0_HiC
Cavia porcellus	64	Caviidae	2.7	3.144	27.9	Cavpor3.0
Chinchilla lanigera	64	Chinchillidae	2.4	2.846	74.4	ChiLan1.0_HiC
Heterocephalus glaber	-	Heterocephalidae	2.6	4.229	20.5	HetGla_female_1.0
Octodon degus	58	Octodontidae	3	7.135	12.1	OctDeg1.0
*Oryctolagus cuniculus**	44	Leporidae	2.7	N/A	-	OryCun2.0
Ochotona princeps	68	Ochotonidae	2.2	10.421	26.9	OchPri3.0
*Homo sapiens**	46	Hominidae	3.3	N/A	-	GRCh38.p12

* denotes a species assembled to chromosome level, the remainder are assembled to scaffold level.

Supplementary Table 1: Genome assemblies used in the analysis. N/A, do not apply. Related to Figure 1.

Zscore

	Num	Median	Genes	LINEs	LTRs	ERVs
**EBRs**	232	21502	7.456	2.663	-1.679	4.575
**Recent ancestor EBRs**	134	2	5.167	1.305	-1.514	3.293
**Old ancestor EBRs**	44	134615	3.431	2.575	0.243	2.268
**Mouse EBRs**	54	122077	3.476	0.782	-0.948	2.131
**Inversion EBRs**	128	14427	4.787	1.882	-1.552	3.056
**Non-Inversion EBRs**	104	99638	5.814	2.047	-1.026	3.514

pvalue

	Num	Median	Genes	LINEs	LTRs	ERVs
**EBRs**	232	21502	0.001	0.003	0.041	0.001
**Recent ancestor EBRs**	134	2	0.001	0.0979	0.0709	0.002
**Old ancestor EBRs**	44	134615	0.002	0.006	0.51	0.016
**Mouse EBRs**	54	122077	0.01	0.19	0.22	0.014
**Inversion EBRs**	128	14427	0.001	0.031	0.0569	0.007
**Non-Inversion EBRs**	104	99638	0.001	0.022	0.1848	0.001

Supplementary Table 2: RegioneR permutation tests. Association between EBRs and transposable elements (10,000 permutation test $p < 0.01$, Z-score > ± 2.58, two-sided). Related to Figure 1.

Accession code	Reference	SRA library	Mark	Cell type
ERX1946916	Grégoire *et al* 2018	ERR1886418.1	DSBs	steps 1-9 spermatids
ERX1946917	Grégoire *et al* 2018	ERR1886419.1	DSBs	steps 1-9 spermatids
ERX1946918	Grégoire *et al* 2018	ERR1886420.1	DSBs	steps 15-16 spermatids
SRX207541	Erkek *et al* 2013	SRR625509	input for DSB data	sonicated sperm DNA
SRX332343	Hammoud *et al* 2014	SRR948796.1/SRR9487997.1/SRR948798.1	H3K4me3	Spermatogonia
SRX332344	Hammoud *et al* 2014	SRR948799.2	H3K4me3	Spermatocytes
SRX332345	Hammoud *et al* 2014	SRR948800.2	H3K4me3	Spermatids
SRX332346	Hammoud *et al* 2014	SRR948801.1/SRR948802.1/SRR948803.1	H27me3	Spermatogonia
SRX332347	Hammoud *et al* 2014	SRR948804.2	H27me3	Spermatocytes
SRX332348	Hammoud *et al* 2014	SRR948805.2	H27me3	Spermatids
SRX332351	Hammoud *et al* 2014	SRR948808.1/SRR948809.1	H3K27ac	Spermatogonia
SRX332352	Hammoud *et al* 2014	SRR948810.2	H3K27ac	Spermatocytes
SRX332353	Hammoud *et al* 2014	SRR948811.2	H3K27ac	Spermatids
SRX332361	Hammoud *et al* 2014	SRR948821.1/SRR948822.1/SRR948823.1	input	Spermatogonia
SRX332362	Hammoud *et al* 2014	SRR948824.2	input	Spermatocytes
SRX332363	Hammoud *et al* 2014	SRR948825.2	input	Spermatids
SRX3114879	Maezawa *et al* 2018	SRR5956512	ATAC-seq	Spermatocytes
SRX3114880	Maezawa *et al* 2018	SRR5956513	ATAC-seq	Spermatocytes
SRX3114883	Maezawa *et al* 2018	SRR5956516	ATAC-seq	Spermatids
SRX3114884	Maezawa *et al* 2018	SRR5956517	ATAC-seq	Spermatids

Supplementary Table 3: Histone marks, DSBs and ATAC-seq fastq files used in this study. Related to Figure 2, 3 and 4.

State	Spermatogonia			Spermatocytes			Spermatids		
	% Coverage	Max (bp)	Average (bp)	% Coverage	Max (bp)	Average (bp)	% Coverage	Max (bp)	Average (bp)
**E1**	1.37	72200	6537	8.01	234200	11895	48.97	281800	11910
**E2**	1.1	35200	2129	2.23	61200	3282	26.39	121000	4876
**E3**	1.18	62000	4560	78.57	769600	24808	10.62	120600	6208
**E4**	0.31	28000	2186	4.44	40200	1632	5.11	95200	1968
**E5**	11.48	23200	611	0.05	4400	534	0.01	3400	550
**E6**	0.07	4000	669	0.88	11400	992	2.94	38600	1802
**E7**	82.3	3103000	4216	3.67	3106800	20900	3.05	3109400	50753
**E8**	2.19	16600	1827	2.15	23400	1882	2.91	20400	1379

Supplementary Table 4: ChromHMM emission state E1-E8 statistics. Related to Figure 2, 3 and 4.

Three-cell type states	Mean (bp)	Median	Max	% Coverage
E0-E0-E0	3.39	1.8	3,102,800	54,467
E0-E0-E2	2.424	1.4	71400	21,841
E5-E0-E0	508	400	9800	5,991
E5-E0-E2	490	400	6600	2,233
other	527	400	23	1,890
E0-E0-E4	810	600	17.6	1,806
E0-E0-E8	886	600	11.4	1,495
E0-E4-E4	722	600	11.8	1,075
E8-E8-E6	1.653	1.4	14.4	1,005
E5-E4-E4	754	600	10.4	0.96
E0-E2-E2	1.988	1200	24	0.928
E0-E2-E0	1.661	1200	20.6	0.751
E0-E4-E0	723	600	9.6	0.704
E5-E0-E4	522	400	7.4	0.65
E0-E4-E2	766	600	9	0.492
E5-E4-E0	605	400	6.4	0.359
E2-E0-E2	1.5	1000	12.8	0.313
E5-E0-E8	476	400	3.8	0.291
E8-E6-E6	941	800	6.8	0.284
E5-E6-E6	589	400	6.6	0.212
E0-E8-E6	558	400	4.8	0.211
E8-E8-E8	534	400	7	0.197
E0-E0-E6	467	400	3.4	0.194
E0-E8-E8	510	400	3.8	0.188
E8-E0-E8	522	400	5.4	0.177
E2-E0-E8	996	800	9.2	0.154
E5-E4-E6	438	400	4	0.149
E5-E4-E2	510	400	4.8	0.141
E0-E6-E6	473	400	3.2	0.137
E2-E8-E6	820	600	6.4	0.127
E8-E0-E2	492	400	4.2	0.12
E4-E4-E4	1.622	1000	12.8	0.118
E0-E4-E6	395	400	3	0.11
E5-E8-E6	555	400	4.4	0.101
E0-E2-E8	708	600	6.6	0.1
E6-E6-E6	523	400	2.8	0.029

* minimum three-cell type state size, was 200bp for all states (the default resolution of chromHMM)

Supplementary Table 5: Summary of the 35 three-cell type states. Related to Figure 2, 3 and 4.

	EBRs		Cohesins	
	**pvalue**	**Zscore**	**pvalue**	**Zscore**
**EBRs**	0.00009999	933.0038	0.00009999	-5.9644
**Cohesins**	0.00009999	-5.2885	0.00009999	1361.3839
**E8-E8-E6 with cohesins**	0.05319468	0	0.00009999	632.6484
**E8-E8-E6 without cohesins**	0.00009999	32.7191	0.00009999	-10.0971
**E0-E6-E6 with cohesins**	0.25287471	0	0.00009999	499.5403
**E0-E6-E6 without cohesins**	0.00009999	9.6461	0.00009999	-6.0892
**E5-E4-E6 with cohesins**	0.30836916	0	0.00009999	197.218
**E5-E4-E6 without cohesins**	0.00039996	3.6905	0.00009999	-7.4785

Supplementary Table 6: Multipermutation tests. Association between EBRs, open chromatin transitions and cohesins (10,000 permutation test $p < 0.01$, Z-score > ± 2.58, two-sided). Related to Figure 2.

					Per chromosome			
**Chr**	**Start**	**End**	**ID Region**	**Lenght (bp)**	**Total Regions**	**Mean Lenght (Mb)**	**Min Lenght (Mb)**	**Max Lenght (Mb)**
1	83750000	86500000	1a	750000	4	1.12	0.75	2.05
1	100800000	104850000	1b	2050000				
1	116350000	119400000	1c	1050000				
1	111350000	114350000	1d	1000000				
3	73550000	76050000	3a	500000	2	0.5	0.5	0.5
3	90100000	92650000	3b	550000				
4	59100000	62200000	4a	1100000	4	0.9	0.5	2
4	60400000	63000000	4b	600000				
4	72550000	75050000	4c	500000				
4	111150000	115150000	4d	2000000				
5	24700000	27200000	5a	500000	5	0.73	0.5	1.8
5	13800000	16650000	5b	850000				
5	92500000	96300000	5c	1800000				
5	108000000	110500000	5d	500000				
5	144550000	147100000	5e	550000				
6	56800000	59300000	6a	500000	2	0.5	0.5	0.5
6	67450000	69950000	6b	500000				
7	46350000	49850000	7a	1500000	4	0.78	0.5	1.5
7	57850000	62950000	7b	500000				
7	61450000	63950000	7c	500000				
7	83950000	86950000	7d	1000000				
8	50550000	53550000	8a	1000000	2	0.7	0.5	1
8	70500000	73000000	8b	500000				
9	18700000	21200000	9a	500000	2	0.5	0.5	0.5
9	34800000	37300000	9b	500000				
12	102700000	105200000	12a	500000	2	0.86	0.5	1.5
12	113250000	116750000	12b	1500000				
13	21750000	24300000	13a	550000	3	0.52	0.5	0.55
13	32300000	34800000	13b	500000				
13	67050000	69550000	13c	500000				
17	17850000	20350000	17a	500000	2	0.88	0.5	1.55
17	37400000	40950000	17b	1550000				
19	6650000	9650000	19a	1000000	4	0.84	0.5	1
19	8150000	10650000	19b	500000				
19	11650000	14650000	19c	1000000				
19	37950000	40950000	19d	1000000				

Supplementary Table 7: Intra-LRIs regions detected in round spermatids. Related to Figures 4 and 5.

Chr	Start	End	ID Region	Interaction (ID region)	Distance Regions Interaction (Mb)	Total interactions per Chr
1	84750000	85500000	1a	1c	33.8	3
1	101800000	103850000	1b	1c/1d	16.1/16.8	
1	117350000	118400000	1c	1a/1b	33.8/16.1	
1	112350000	113350000	1d	1b	16.8	
3	74550000	75050000	3a	3b	17.3	1
3	91100000	91650000	3b	3a	17.3	
4	60100000	61200000	4a	4c/4d	14.1/54.2	2
4	61400000	62000000	4b	4d	54.2	
4	73550000	74050000	4c	4a	14.1	
4	112150000	114150000	4d	4a/4b	54.2	
5	25700000	26200000	5a	5b	11.6	3
5	14800000	15650000	5b	5a	11.6	
5	93500000	95300000	5c	5d/5e	16.2/52.8	
5	109000000	109500000	5d	5c	16.2	
5	145550000	146100000	5e	5c	52.8	
6	57800000	58300000	6a	6b	11.3	1
6	68450000	68950000	6b	6a	11.3	
7	47350000	48850000	7a	7c	15.8	2
7	58850000	61950000	7b	7d	26.8	
7	62450000	62950000	7c	7a	15.8	
7	84950000	85950000	7d	7b	26.8	
8	51550000	52550000	8a	8b	22.7	1
8	73550000	74050000	8b	8a	22.7	
9	19700000	20200000	9a	9b	16.8	1
9	35800000	36300000	9b	9a	16.8	
12	103700000	104200000	12a	12b	12.2	1
12	114250000	115750000	12b	12a	12.2	
13	22750000	23300000	13a	13b	11.2	2
13	33300000	33800000	13b	13a/13c	11.2/35.4	
13	68050000	68550000	13c	13b	35.4	
17	18850000	19350000	17a	17b	21.3	1
17	38400000	39950000	17b	17a	21.3	
19	7650000	8650000	19a	19d	35.4	4
19	9150000	9650000	19b	19d	31	
19	12650000	13650000	19c	19d	27.5	
19	38950000	39950000	19d	19a/19b/19c	35.4/31/27.5	

Supplementary Table 8: Interactions between intra-LRIs regions detected in round spermatids. Related to Figures 4 and 5.
