# Supplementary material for: 3D chromatin remodelling in the germ line modulates genome evolutionary plasticity
Source: Nat Commun. 2022 May 11;13:2608. doi: 10.1038/s41467-022-30296-6 (PMC9095871; doi:10.1038/s41467-022-30296-6)
Supplement: Supplementary file 2 — Description of additional Supplementary Files [file 41467_2022_30296_MOESM2_ESM.pdf]

Descriptions of Additional Supplementary Data Files

Supplementary Data 1 - GO terms of genes located in intra-LRIs. The raw p-values are determined by Fisher's exact test and the False Discovery Rate as calculated by the Benjamini-Hochberg procedure. Enrichment Score (ES) is calculated by DAVID algorithm. Related to Figures 4 and 5.

Supplementary Data 2 - Inter-LRIs regions detected in round spermatids. Related to Figures 4 and 5.

Supplementary Data 3 - GO terms of genes located in inter-LRIs. The raw p-values are determined by Fisher's exact test and the False Discovery Rate as calculated by the Benjamini-Hochberg procedure. Enrichment Score (ES) is calculated by DAVID algorithm. Related to Figures 4 and 5.

Supplementary Data 4 - List of ancestral syntenic associations with LRIs detected in the mouse genome. Related to Figures 4 and 5.
